# Transcriptional Response of Two *Brassica napus* Cultivars to Short-Term Hypoxia in the Root Zone

**DOI:** 10.3389/fpls.2022.897673

**Published:** 2022-04-29

**Authors:** Stefanie Ambros, Mona Kotewitsch, Philipp R. Wittig, Bettina Bammer, Angelika Mustroph

**Affiliations:** Department of Plant Physiology, University of Bayreuth, Bayreuth, Germany

**Keywords:** *Brassica napus*, waterlogging, root-zone hypoxia, fermentation, RNA sequencing

## Abstract

Waterlogging is one major stress for crops and causes multiple problems for plants, for example low gas diffusion, changes in redox potential and accumulation of toxic metabolites. *Brassica napus* is an important oil crop with high waterlogging sensitivity, which may cause severe yield losses. Its reactions to the stress are not fully understood. In this work the transcriptional response of rapeseed to one aspect of waterlogging, hypoxia in the root zone, was analyzed by RNAseq, including two rapeseed cultivars from different origin, Avatar from Europe and Zhongshuang 9 from Asia. Both cultivars showed a high number of differentially expressed genes in roots after 4 and 24 h of hypoxia. The response included many well-known hypoxia-induced genes such as genes coding for glycolytic and fermentative enzymes, and strongly resembled the hypoxia response of the model organism *Arabidopsis thaliana*. The carbohydrate status of roots, however, was minimally affected by root hypoxia, with a tendency of carbohydrate accumulation rather than a carbon starvation. Leaves did not respond to the root stress after a 24-h treatment. In agreement with the gene expression data, subsequent experiments with soil waterlogging for up to 14 days revealed no differences in response or tolerance to waterlogging between the two genotypes used in this study. Interestingly, using a 0.1% starch solution for waterlogging, which caused a lowered soil redox potential, resulted in much stronger effects of the stress treatment than using pure water suggesting a new screening method for rapeseed cultivars in future experiments.

## Introduction

Many crop plants are very sensitive to flooding periods which may occur after heavy rainfall, rising sea water, or during fast snow melting in spring. Besides other effects, flooding severely restricts gas diffusion into and out of the plant tissues that are under water, leading to deficiency in oxygen and carbon dioxide. The increased frequency of extreme weather events due to climate change affects certain regions around the world, and these regions at risk might expand in the future even more (e.g., Kundzewicz et al., 2014; Trenberth et al., 2014; Pekel et al., 2016; Blöschl et al., 2019). Flooding events can be differentiated into two types that require different adaptations of plants, namely full submergence and root waterlogging (Sasidharan et al., 2017).

Waterlogging is considered as the lesser problematic stress type. Many plants have developed adaptational mechanisms to cope with this type of stress, resulting in an avoidance or a tolerance strategy. One very important response to waterlogging is the formation of aerenchyma tissue within leaves, stems and roots, thus avoiding oxygen deficiency within plant organs that are under water (summarized in Voesenek and Bailey-Serres, 2015; Mustroph et al., 2018; Yamauchi et al., 2018). Other mechanisms such as a barrier against radial oxygen loss in underwater organs and the formation of adventitious roots with high porosity also help to maintain a sufficient oxygen content within plant tissues.

Besides these avoidance mechanisms, there are also acclimation responses that enable tolerance to low oxygen concentrations within plant tissues at least for some time (summarized in Bailey-Serres et al., 2012; Voesenek and Bailey-Serres, 2015; Mustroph et al., 2018). These mainly include biochemical modifications such as induction of glycolysis and fermentation in order to maintain energy production through periods with limited mitochondrial respiration. These responses also include specific enzymes or isoforms of starch and sucrose cleavage and glycolytic by-passes (summarized in Huang et al., 2008; Mustroph et al., 2014, 2018; Atwell et al., 2015). Furthermore, growth and biosynthetic processes are strongly down-regulated under low-oxygen stress, including ribosomal activity on non-essential transcripts, since translation is an energy-consuming process (Branco-Price et al., 2008; Mustroph et al., 2009).

However, most crop species cannot tolerate longer periods of waterlogging since they only possess a limited acclimation potential. *Brassica napus* is an important oil crop plant and it is also used for animal feed. However, this plant species is very sensitive to soil flooding and waterlogging, since it is not able to form aerenchyma in the roots and exhibits a high radial oxygen loss under water (Voesenek et al., 1999). Still, rapeseed is often used as a rotation crop on rice fields in Asia (Zou et al., 2013a; Lee et al., 2014), and therefore is often subjected to flooding conditions. Despite the importance and sensitivity of this crop, little is known about its molecular response to flooding conditions such as waterlogging and submergence. Such information could be vital to develop cultivars with enhanced flooding tolerance.

Indeed, several approaches have been used to identify rapeseed cultivars with higher waterlogging tolerance within the last years, mainly focusing on Asian cultivars (e.g., Zou et al., 2013b,^2014^; summarized in Mustroph, 2018). However, the molecular bases for these tolerance traits are still not known, despite efforts to study the response of different cultivars at the level of transcriptome (Zou et al., 2013a,^2015^) and proteome (Xu et al., 2018). Mapping processes of these cultivars with contrasting responses are ongoing, with no clear results so far (Ding et al., 2020). There could be several reasons for it. For example, the molecular analyses were performed at specific developmental stages or with a stress duration that did not reveal differences between the cultivars. The other possibility would be that little genetic differences in waterlogging tolerance exist between genotypes studied so far, despite interesting observations in the field. We therefore raised the following hypothesis: if differences in waterlogging tolerance between contrasting rapeseed cultivars exist, those should emerge through a comparison of an Asian cultivar with observed waterlogging tolerance, Zhongshuang 9 (Zou et al., 2013b,^2014^), and a European cultivar not bred for flooding tolerance.

There is evidence that *Brassica napus* roots can respond to waterlogging and the associated hypoxia at the transcriptional level, for example by induction of genes coding for fermentative enzymes (Zou et al., 2013a,^2015^), while leaves above air responded in a different way (Lee et al., 2014). On the other hand, avoidance mechanisms such as the formation of aerenchyma are not present in this species (Voesenek et al., 1999; Ploschuk et al., 2018). However, the existing transcriptome analyses were performed without the full genome information on *Brassica napus*, which was only published later (Chalhoub et al., 2014). It is therefore not known whether all or only a few gene copies for one gene function of the tetraploid species are hypoxia responsive. We hypothesize that most or all gene copies of a hypoxia-induced gene in the rapeseed genome are responsive to the stress treatment, which would require modification of not only one but multiple target genes by breeders.

The aim of our work was to (1) compare the transcriptional responses of two different *Brassica napus* cultivars to one aspect of waterlogging, hypoxic conditions in the root zone, including the analysis of roots and leaves at two different time points and (2) to evaluate with different growth conditions whether a difference in waterlogging tolerance exists between the two cultivars. In our previous analysis utilizing the same two cultivars (Wittig et al., 2021), we could not identify significant differences in submergence tolerance, but a strong transcriptional response to submergence in leaves of both genotypes. Here, we also identified a large number of transcripts induced by hypoxia in roots, which were mainly similar between the cultivars and also similar to the transcriptional response of *Arabidopsis thaliana* to hypoxia (Mustroph et al., 2009, 2010; Lee and Bailey-Serres, 2019). However, the response of hypoxic roots was very different from submerged rapeseed leaves. In addition, and in accordance with our analysis under submergence, we could not identify differences in waterlogging tolerance between the two cultivars, despite the application of several growth conditions and stress treatment methods.

## Materials and Methods

### Plant Material and Growth Conditions

Seeds of two rapeseed cultivars were used, the hybrid winter cultivar Avatar (Wollmer et al., 2018; Wittig et al., 2021) as well as the semi-winter cultivar Zhongshuang 9 (Zou et al., 2013a; Wittig et al., 2021).

For the experiments with plants in hydroponics, dry seeds were sterilized in a chlorine gas atmosphere for 45 min. After removal of the gas, the sterilized seeds were germinated in small tubes containing 1:10 Hoagland solution (0.28 mM Ca(NO_3_)_2_, 0.1 mM (NH_4_)H_2_PO_4_, 0.2 mM MgSO_4_, 0.6 mM KNO_3_, 5 μM of a complex of Fe(III) and N,N′-di-(2-hydroxybenzoyl)-ethylenediamine-N,N′-diacetate (ABCR, Karlsruhe, Germany), pH 5.7). Plants were grown in a short-day chamber (8 h illumination with ca. 100 μmol photons * m^–2^ * s^–1^) at 23°C. After 4 days, the tubes were transferred into large buckets (4.5 l of volume) by use of a perforated plate. The buckets were filled with KNOP nutrient solution (Mustroph et al., 2006), which was continuously bubbled with air. The nutrient solution was replaced twice a week. When plants were 15 days old, the stress treatment was applied to half of the plants by bubbling the nutrient solution with nitrogen gas for the time indicated. The shoot tissues remained in air.

For the experiments with plants on soil, seeds were pre-germinated in the dark at 30°C for 24 h on moist filter paper. Subsequently, germinating seeds were transferred into pots (5.3 × 5.3 cm) filled with a soil-sand mixture. The soil used was standard potting soil type GS90 coarse: potting soil (Ökohum GmbH): vermiculite in a ratio of 3:3:1. The potting soil was mixed with sand in a ratio of 3:1. Plants were grown in a short-day chamber (8 h illumination with ca. 100 μmol photons * m^–2^ * s^–1^) at 23°C. 15-day-old plants were used for the experiments. For stress treatment of soil-grown plants, two waterlogging variants were used. In a first set, pots were placed in a box, which was subsequently filled with tap water up to the soil surface. In a second set, one portion of the pots were placed into boxes and filled with a 0.1% starch solution in deionized water (Mano and Takeda, 2012; Miricescu et al., 2021), while another portion of the pots were waterlogged with deionized water only. Control pots were watered regularly as needed. Both treatments lasted for two weeks. The number of plants per replicate is specified in the respective figure part.

### RNA Extraction, RNA Sequencing and Bioinformatics

For transcriptome analyses, root and leaf tissue from plants grown in hydroponics were used. Plants were stressed with 4 and 24 h of nitrogen flushing of the medium in the root zone. Subsequently, the entire root system and the first true leaf was harvested together with air-gassed controls and immediately frozen in liquid nitrogen. The frozen tissue was ground in liquid nitrogen. RNA extraction, quality control, processing of the RNA for sequencing, and the subsequent bioinformatics analyses were carried out exactly as previously described (Wittig et al., 2021). Briefly, RNA was extracted by use of the ISOLATE II RNA plant kit (Bioline, Luckenwalde, Germany). After quality controls through gel electrophoresis and fluorimeter measurements, RNA was further processed by Eurofins Genomics Europe Shared Services GmbH (Ebersberg, Germany). Sequencing was done with the 150 bp paired-end mode on the Illumina HiSeq 4000 platform.

Three replicates per time point and genotype were done, resulting in a total of 36 libraries, with 30 Mio to 50 Mio reads per library (Supplementary Table 1). Transcript quantification was done by use of the Kallisto software (Bray et al., 2016). About 76 to 80% of the reads from the cultivar Avatar aligned to the reference genome of Darmor (Chalhoub et al., 2014). Reads from the cultivar Zhongshuang 9 had a mapping rate of 67 to 75% (Supplementary Table 1). Mapping rates for leaves were generally higher than for roots and were similar to the mapping rates in our previous study (Wittig et al., 2021). DEG analysis was carried out with the edgeR and limma Bioconductor packages in R (McCarthy et al., 2012) as previously described (Müller et al., 2021). RNA sequencing raw and processed data have been deposited at the Gene Expression Omnibus database under the accession GSE180262.

### Enzyme Extraction and ADH Activity

Frozen root material from plants grown in hydroponics was ground to a fine powder. Enzymes were extracted in 50 mM Hepes-KOH, pH 6.8 containing 5 mM Mg acetate, 5 mM β-mercaptoethanol, 15% (v/v) glycerin, 1 mM EDTA, 1 mM EGTA, and 0.1 mM Pefabloc proteinase inhibitor (Sigma-Aldrich, Germany). The samples were then centrifuged at 13,000 g at 4°C for 15 min. The resulting supernatant was used for spectrophotometric determination of ADH activity at 340 nm (SPECORD 200 PLUS, Analytic Jena, Germany). The ADH activity was measured in 50 mM TES buffer, pH 7.5 including 0.17 mM NADH (Waters et al., 1991). The reaction was started by adding 10 mM acetaldehyde. The protein content as determined by the Bradford reagent and by use of a BSA standard curve (Bradford, 1976). The number of biological replicates is specified in the respective figure part.

### Carbohydrate Extraction and Measurement of Sugar Content

One leaf of each plant (from hydroponics or soil-grown plants) was frozen in liquid nitrogen and the fresh weight of the samples was determined. The frozen plant samples were ground to a fine powder. To extract soluble sugars, 1 ml of 0.83 N perchloric acid was added, mixed well, and then stored on ice until all samples were processed. The samples were then centrifuged for 15 min at 13,000 g and 4°C, and the supernatant was transferred to a new 1.5 ml Eppendorf reaction tube. The pellet was overlaid with 600 μl of 80% ethanol for subsequent starch extraction and temporarily stored at 4°C. The supernatant was mixed with 200 μl of 1 M Bicine and quickly neutralized with 100 μl of 4 M KOH. The samples were then centrifuged for 10 min at 13,200 rpm and 4°C and the supernatant was transferred to a new reaction tube. The supernatant was either frozen at −20°C or used directly for the measurement of the sugar content.

For starch extraction, the pellet from sugar extraction was mixed with 600 μl of 80% ethanol and centrifuged at 13,000 g and 4°C for 5 min. The supernatant was then removed, and the procedure was repeated once. After removal of the ethanol, 400 μl of 0.2 M KOH was added to the pellet and the samples were homogenized. This was placed on a heating block at 95°C for 1 h. After incubation, the samples were centrifuged for 5 min at 10,000 rpm and 4°C, and the supernatant was transferred to a new 1.5 ml reaction tube. The supernatant was neutralized with 80 μl of 1 N acetic acid. From the extract, 50 μl was then mixed with 100 μl amyloglucosidase (2 mg/ml enzyme in 50 mM sodium acetate pH 5.0) and incubated overnight at 55°C on the heating block.

Both sugar and starch content were measured spectrophotometrically (SPECORD 200 PLUS, Analytic Jena, Germany). For this, 780 μl of 0.1 M imidazole buffer containing 1 mM ATP and 2 mM NAD in a semi-micro cuvette was mixed with 20 μl of each sample and 1 u glucose-6-phosphate dehydrogenase was added. Then, the measurement was started at a wavelength of 340 nm. For both the sugar and the starch measurement 0.5 u of hexokinase in 5 μl of imidazole buffer was added after 10 min to measure glucose. Starch measurement was stopped 20 min after addition of the enzyme. For soluble sugars, 0.2 u phosphoglucose isomerase in 5 μl buffer was pipetted to the samples after a plateau was reached, to measure fructose. After constant values had again been established, 60 u invertase in 5 μl buffer was added and mixed well to determine the sucrose content. The number of biological replicates is specified in the respective figure part.

### Chlorophyll Fluorescence and Chlorophyll Content

To determine the chlorophyll fluorescence, the quantum yield of photosystem II of the plants grown on soil was measured under constant conditions using a Junior-PAM (Walz, Effeltrich, Germany). This PAM measurement (pulse-amplitude-modulated fluorescence measurement) was performed using a saturating pulse method (SAT pulses). A large adult light-adapted leaf of each plant was clamped between two magnets and irradiated for 10 seconds with ambient light of about 100 μmol photons * m^–2^ * s^–1^, and subsequently a saturation pulse was applied. The ΦPSII value was calculated and used for further evaluation.

Determination of chlorophyll content in an invasive way was done as follows. Extraction of chlorophyll was performed according to the protocol of Park et al. (2018). In this process, 1.4 ml of 80% aqueous acetone was added to frozen and ground leaf samples and mixed well. Then, the samples were stored overnight at 4°C. The next day, the leaf samples were centrifuged at 13,000 g for 5 min at 4°C and the supernatant was pipetted into a new tube. For measurement of chlorophyll content, the supernatant was diluted 1:10 with acetone in a semi-micro cuvette and absorbance was measured at 645 nm and 663 nm. Chlorophyll content was calculated by use of the formula developed by Arnon (1949).

Determination of chlorophyll content in a non-invasive way was done by use of the chlorophyll concentration meter MC-100 (Apogee Instruments, Logan, United States) using the settings for kohlrabi.

### Chemical Parameters of the Waterlogging Solution

Oxygen content, oxidation-reduction potential (ORP), pH and conductivity of the waterlogging solution of soil-grown plants was determined by use of the multimeter PCE-PHD 1 (PCE instruments, Meschede, Germany) and the following electrodes: OXPB-11, ORP-14, PE-03, CDPB-03. Measurements were done about 2 to 3 h after start of illumination. The temperature of the waterlogging solution ranged between 19 and 23°C at the time of the measurements.

## Results

### Transcriptome Analysis Under Root Hypoxia

Waterlogging is a complex stress in nature, including limited gas diffusion, microbial activities, changes in redox chemistry and accumulation of toxic metabolites. In a simplified approach we initially focused on the low-oxygen component of waterlogging and therefore performed an RNAseq analysis of plants in hydroponics with their roots exposed to nitrogen gassing causing hypoxia (Supplementary Table 1). This experimental set-up also allowed for controlled aeration of the root system and allowed a direct comparison to the hypoxia response of the model species *Arabidopsis thaliana* (Mustroph et al., 2009).

**FIGURE 1:**
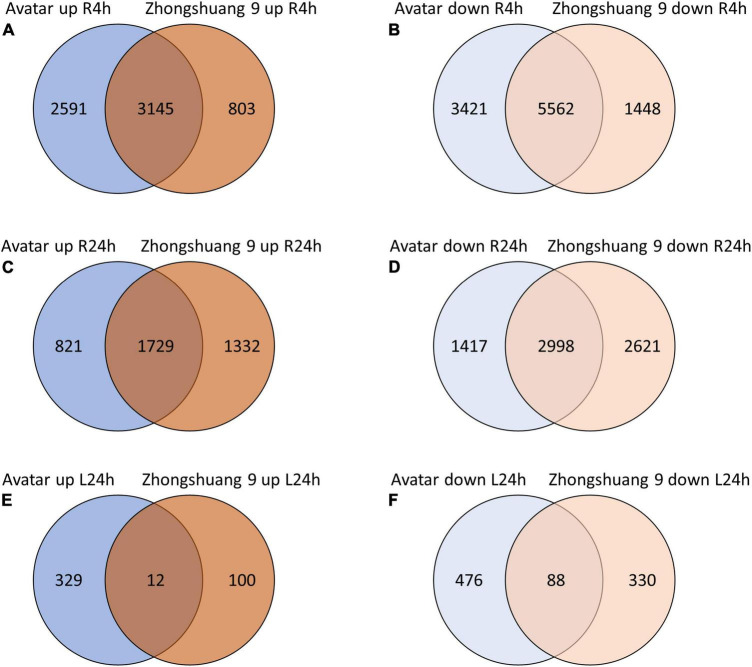
Venn diagrams showing overlaps of induced **(A,C,E)** or repressed **(B,D,F)** genes between two *Brassica napus* cultivars (Avatar, Zhongshuang 9) in hydroponics after 4 and 24 h of root hypoxia, compared with aerated controls. **(A,B)** Roots were treated with 4 h of hypoxia; **(C,D)** Roots were treated with 24 h of hypoxia; **(E,F)** Leaves of plants treated with root hypoxia for 24 h.

Treating plant roots with hypoxia caused a strong transcriptional response in roots of both genotypes. After 4 h, 5,736 and 3,948 transcripts were significantly up-regulated compared to air-treated controls (log2FC > 1, FDR < 0.01) in Avatar and Zhongshuang 9, respectively, with an overlap of 3,145 transcripts (Figure 1 and Supplementary Table 2, columns R to AD). After 24 h, 2,550 and 3,061 transcripts were induced, with an overlap of 1,729 transcripts. The overlap between 4 h and 24 h was 1,897 and 1,844 transcripts for Avatar and Zhongshuang 9, respectively (Supplementary Figure 1). The number of down-regulated genes in roots was even higher. 8,983 and 7,010 transcripts were down-regulated after 4 h, and 4,415 and 5,619 transcripts were down-regulated after 24 h in Avatar and Zhongshuang 9, respectively. Again, there was a high similarity between the time points with 2,952 and 3,237 transcripts commonly downregulated in Avatar and Zhongshuang 9, respectively (Supplementary Figure 1). The response of the leaves to hypoxia in the root zone was only analyzed after 24 h and was much lower, with 341 and 112 transcripts significantly induced in Avatar and Zhongshuang 9, with an overlap of only 12 transcripts. 564 and 418 transcripts were significantly down-regulated in leaves, with an overlap of 88 transcripts (Figure 1 and Supplementary Table 2, columns R to AD).

### Roots Induce a Typical and Strong Hypoxia Response

A functional gene ontology (GO) analysis of the transcripts of roots responding to nitrogen-gassing revealed many GO categories to be significantly enriched (Supplementary Table 3). After 4 h, many categories involved in stress response were enriched, for example “response to chitin,” “heat acclimation,” “respiratory burst involved in defense response,” and “intracellular signal transduction”. Furthermore, hormonal responses were induced, such as “response to ethylene,” “response to jasmonic acid,” and “salicylic acid mediated signaling pathway.” The hypoxia-related terms “response to hypoxia” and “anaerobic respiration” were enriched, and this enrichment was even higher after 24 h. Comparison of genotypes revealed a very similar response, but surprisingly, a few chloroplast-related GOs were enriched only in Avatar roots after 4 h (Supplementary Table 3).

In general, the response in roots after 24 h was very similar to the response after 4 h, but fewer genes remained induced (Supplementary Figure 1). The functional categories “jasmonic acid biosynthetic process,” “response to water deprivation,” and “endoplasmic reticulum unfolded protein response” were less enriched after 24 than after 4 h (Supplementary Table 3).

Among down-regulated genes after 4 h, we observed the functional categories “extracellular region,” “anchored component of membrane,” “cell proliferation,” “histone H3-K9 methylation,” “plant-type cell wall,” and categories associated with DNA replication. DNA replication was still enriched in Zhongshuang 9 after 24 h, but much less affected in Avatar after 24 h. In contrast, some categories, for example “trichoblast differentiation”, were more enriched in Avatar after 4 and 24 h than in the other genotype. Furthermore, “Casparian strip” was more enriched after 24 than 4 h in both genotypes. Among enriched GO terms of down-regulated genes, many were associated with biosynthetic processes (Supplementary Table 3).

**FIGURE 2:**
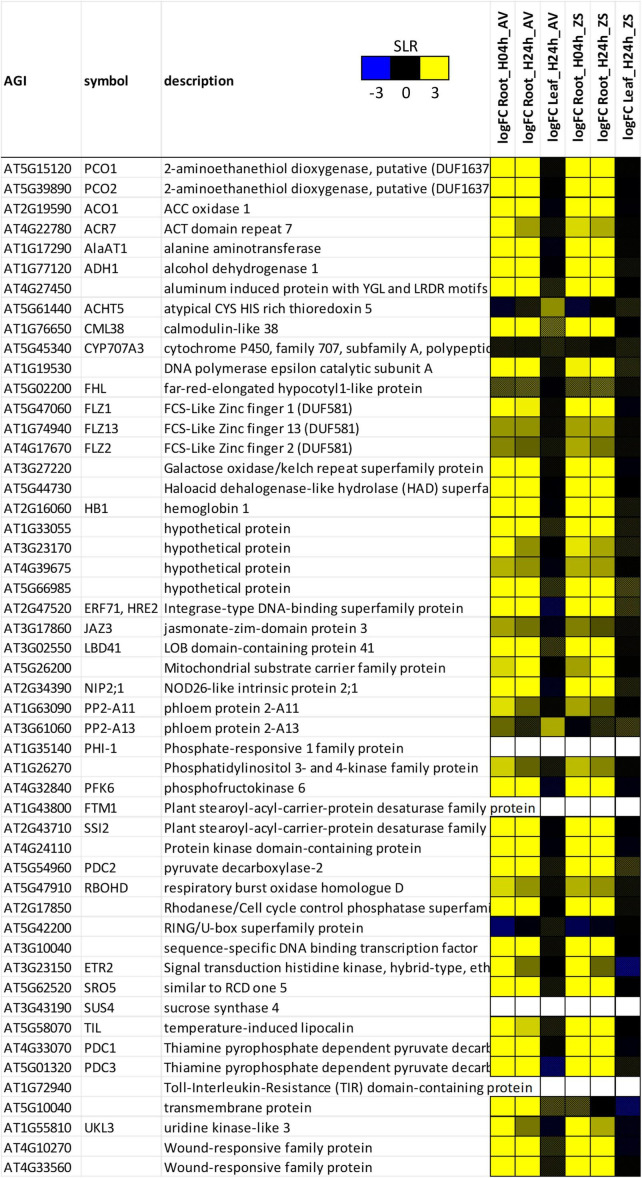
Heatmap of hypoxia core-response genes (Mustroph et al., 2009) from the *Brassica napus* expression data. A summary of up to six rapeseed gene IDs for one Arabidopsis gene ID is shown. For expression of all rapeseed genes, please see Supplementary Figure 2. Values are signal-log ratios (SLR) of summed counts of root-zone hypoxia vs. aerated control. AV, Avatar; ZS, Zhongshuang 9. The color intensity reflects the SLR values (blue, –3; yellow, + 3). Crossed cells represent values that are not significant (FDR > 0.01).

The enrichment of hypoxia-related categories in this dataset let us to compare the rapeseed hypoxia response to the previously defined hypoxia core response genes (HRGs) of Arabidopsis (Mustroph et al., 2009). Indeed, of the 49 HRGs from Arabidopsis (Figure 2), which correspond to 161 expressed transcripts in rapeseed (Supplementary Figure 2), 118 to 127 transcripts responded to hypoxia in roots after 4 and 24 h, and therefore showed a highly significant enrichment (Supplementary Table 2, column Q; Supplementary Table 4). In many cases, most or all isogenes of one HRG in Arabidopsis responded in a similar manner to the stress in rapeseed, but with some exceptions, for example *ACT DOMAIN REPEAT* 7 (*ACR7*), *ALANINE AMINTRANSFERASE 1* (*AlaAT1*), *FCS-LIKE ZINC FINGER PROTEINS with DUF581* (*FLZ1*, *FLZ2* and *FLZ13*), *AT4G39675* (hypothetical protein), *PHLOEM PROTEIN 2-A FAMILY* (*PP2-A11* and *PP2-A13*), and *RESPIRATORY BURST OXIDASE HOMOLOGUE D* (*RBOHD*) (Supplementary Figure 2). Both genotypes responded in a very similar way, and only one HRG, *AT5G10040*, was induced by hypoxia in Avatar, but not in Zhongshuang 9 (Figure 2 and Supplementary Figure 2).

Of the genes highly expressed in hypoxic rapeseed roots, several have a known function under hypoxia, for example primary metabolism and glycolysis (*PYRUVATE DECARBOXYLASE*, *PDC1* and *PDC3*; *ALCOHOL DEHYDROGENASE 1*, *ADH1*; *AlaAT1*; *SUCROSE SYNTHASE 1*, *SUS1*; *FRUCTOKINASE 2*, *FRK2*; *PHOSPHOFRUCTOKINASE*, *PFK3* and *PFK6*; *FRUCTOSE-BISPHOSPHATE ALDOLASE 6*, *FBA6*), or signal transduction (*HYPOXIA-RESPONSIVE ERF*, *HRE1* and *HRE2*; *HYPOXIA RESPONSE ATTENUATOR 1*, *HRA1*; *LOB DOMAIN CONTAINING PROTEIN 41*, *LBD41*). A few HRGs were not induced by hypoxia in rapeseed, but in Arabidopsis (*ATYPICAL CYS HIS RICH THIOREDOXIN*, *ACHT5*; *ABSCISIC ACID 8’-HYDROXYLASE*, *CYP707A3*; *FAR-RED-ELONGATED HYPOCOTYL1-LIKE PROTEIN*, *FHL*, and a RING/U-box superfamily protein, *AT5G42200*).

Interestingly, there was also an overlap of hypoxia-down-regulated genes between Arabidopsis and rapeseed (Supplementary Table 2, column P; Supplementary Table 4), for example several genes coding for invertase/pectin methylesterase inhibitor superfamily proteins, nodulin MtN21/EamA-like transporter family proteins (*UMAMIT12*, *UMAMIT17*), as well as several genes coding for biosynthesis enzymes (e.g., 3-ketoacyl-CoA synthase 20, *KCS20*; cytochrome P450 family proteins *CYP83B1*, *CYP82F1*, *CYP79B2*; Galactose mutarotase-like superfamily protein).

### Transcriptional Changes of Roots in Primary Metabolism, Plant Hormones and Starvation-Responses

The functional categorization by use of GO terms (Supplementary Table 3) suggested a transcriptional response of several pathways to root-zone hypoxia, including primary metabolism and hormone-associated pathways. This let us to have a deeper look into specific pathways. In primary metabolism, the expression of many genes coding for enzymes in glycolysis and fermentation were up-regulated, including hexokinase, phosphofructokinase and aldolase (Supplementary Table 5). For sucrolysis, sucrose-synthase-coding genes were induced, while invertase-like genes were reduced in their expression. Pyruvate dehydrogenase, TCA-cycle enzymes and enzymes for the pentose-phosphate cycle were not modified in expression or even down-regulated. Interestingly, some alternative enzymes were significantly induced in Avatar at the transcript level after 4 h of hypoxia, namely *ISOCITRATE LYASE* from the glyoxylate cycle, and *PYRUVATE ORTHOPHOSPHATE DIKINASE* (*PPDK*) from gluconeogenesis. Among genes coding for nitrogen metabolism, several aminotransferases were induced in both cultivars, for example isoforms for *AlaAT1* and *ASPARTATE AMINOTRANSFERASE 2* (*AspAT2*) (Supplementary Table 5).

Plant hormone biosynthesis and signaling were also among the enriched GO categories within the differentially expressed genes in roots under hypoxia (Supplementary Table 3). While auxin biosynthesis was not clearly modified, several polar auxin transporters were negatively affected after 4 h of hypoxia. Despite this, several members of the SAUR-like auxin-responsive protein family were differentially expressed, but with no clear trend (Supplementary Table 6). Cytokinin biosynthesis and signaling were in tendency negatively affected by root hypoxia (Supplementary Tables 3, 6), with the exception of the HPt factor gene *HPT PHOSPHOTRANSMITTER 4* (*AHP4*), whose expression was strongly induced after 4 and 24 h of hypoxia. In gibberellin metabolism, some genes coding for enzymes involved in biosynthesis and degradation were differentially expressed, but the respective GO terms were not enriched. Signaling related to this group of plant hormones was not affected by hypoxia (Supplementary Tables 3, 6). Brassinosteroid biosynthesis, on the other hand, was negatively regulated by hypoxia.

Among the stress-related hormones, there was a strong enrichment of genes responding to either ethylene or abscisic acid (Supplementary Table 3). However, while neither abscisic acid biosynthesis nor degradation was strongly affected by the stress, genes coding for biosynthetic enzymes in ethylene production were induced upon hypoxia (*1-AMINO-CYCLOPROPANE-1-CARBOXYLATE SYNTHASE 2* and *1-AMINO-CYCLOPROPANE-1-CARBOXYLATE OXIDASE 1*, Supplementary Table 6). Several genes coding for different steps of jasmonic acid biosynthesis and signal transduction were induced especially after 4 h of root hypoxia, and parts of the salicylic acid signal transduction were induced after 4 and 24 h of hypoxia (Supplementary Table 3).

Previously we have shown that submerged leaves of *Brassica napus* showed a strong starvation response (Wittig et al., 2021). We therefore compared the present dataset to the starvation-responsive genes of Arabidopsis (Usadel et al., 2008; Cookson et al., 2016). Indeed, there was an enrichment of starvation-induced genes (Supplementary Table 4), although less pronounced than under submergence, and affecting different genes. Among starvation-responsive genes in roots under hypoxia, the following transcripts were found: *CHY-type/CTCHY-type/RING-type ZINC FINGER PROTEIN* (*AT5G22920*), Eukaryotic aspartyl protease family protein (*AT5G19120*), *GLUTAMINE-DEPENDENT ASPARAGINE SYNTHASE 1* (*ASN1*), and *MATERNAL EFFECT EMBRYO ARREST 14* (*MEE14*) (Supplementary Table 2, columns R to AD).

### Biochemical Modifications in Response to Root-Zone Hypoxia

**FIGURE 3:**
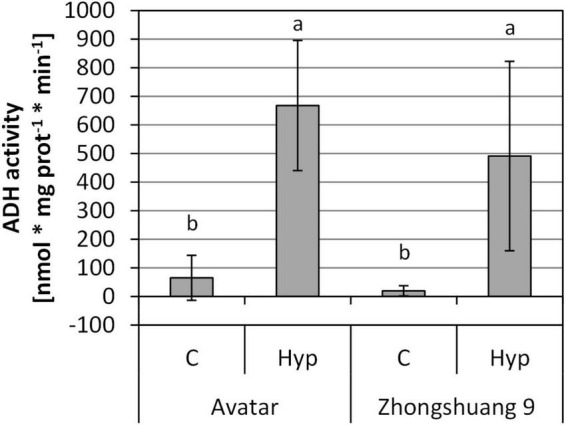
ADH activity [nmol * mg prot^–1^ * min^–1^] in roots of two *Brassica napus* cultivars (Avatar, Zhongshuang 9) in hydroponics after 3 days of root hypoxia, compared with aerated controls. At time of harvest, plants were 18 days old. Data are means ± SD of 4 to 5 replicates. Different letters indicate significant differences (ANOVA and Tukey *post hoc* test, *p* < 0.05).

The transcriptional data suggest an induction of transcripts coding for fermentative enzymes (Supplementary Table 2, columns R to AD, Supplementary Table 3). In order to analyze whether these transcriptional changes translate into an enhanced activity of fermentative enzymes, we measured the activity of ADH after hypoxic treatment in the root zone. After 4 and 24 h we did not observe any significant increase in ADH activity (Supplementary Figure 3). However, after 3 days of stress, a strong and significant induction of ADH activity was observed in roots of both cultivars, Avatar and Zhongshuang 9, although no differences were detected between the genotypes (Figure 3). This indicates a strong hypoxia response also at the protein level, albeit with a delay in comparison to the transcriptional changes.

**FIGURE 4:**
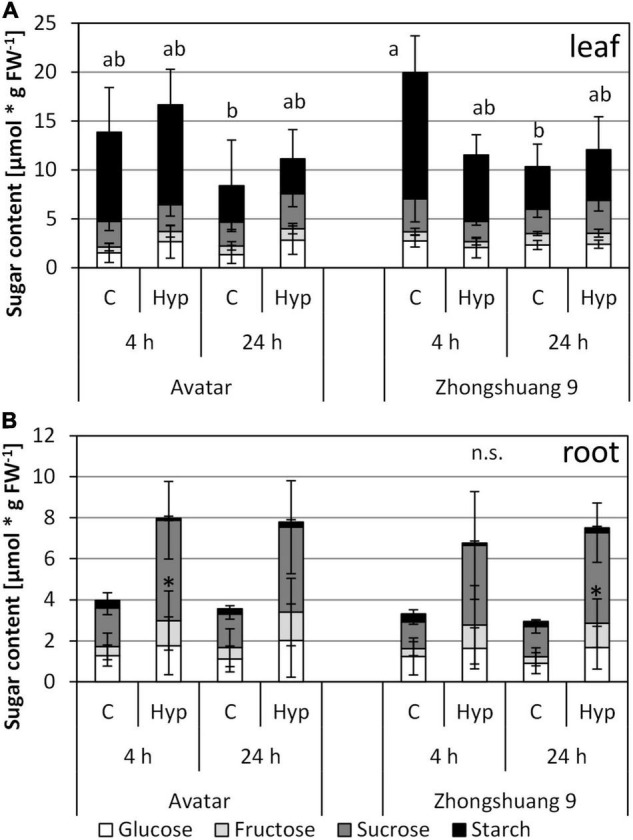
Content of carbohydrates [μmol * g FW^–1^] in leaves **(A)** and roots **(B)** of two *Brassica napus* cultivars (Avatar, Zhongshuang 9) in hydroponics after 4 and 24 h of root hypoxia, compared with aerated controls. At time of start of stress, plants were 15 days old. Data are means ± SD of 5 replicates. Different letters indicate significant differences for the sum of sugars (ANOVA and Tukey *post hoc* test, *p* < 0.05). n.s., ANOVA was not significant; *, significant compared with respective control (*T*-Test, *p* < 0.05).

Several starvation-responsive genes were among the hypoxia-induced genes in roots after 4 and 24 h (Supplementary Table 2, columns M and N), but the enrichment was much lower (Supplementary Table 4) than in our previous study on complete submergence. Therefore, the carbohydrate content in roots and leaves was measured after both time points. Interestingly, leaf sugar content did not significantly differ between aeration and root hypoxia, and between genotypes (Figure 4A). There was a slightly lower level of carbohydrates at the 24-h-time point for stress and control samples, since these plants had only 2 h of light after the 16-h-night, while the plants from the 4-h-time point had 6 h of light.

In roots, carbohydrate levels, especially starch, were lower than in leaves under both control and stressed conditions (Figure 4B). In this case, a tendency of higher sugar contents after stress treatment was observed, which was only significant for sucrose. Again, there was no difference between the genotypes. Sugar starvation due to lower sugar content can therefore be excluded. However, the transcriptomic data still suggest a starvation response (Supplementary Table 2, columns M and N). This could be a hint that carbohydrates were not fully available for the plant cells and their metabolism. This assumed lower consumption rate could be an explanation for the observed slight rise in sugar levels in roots.

### Comparison to Previous Rapeseed Transcriptome Analyses Under Flooding-Related Stress Treatments

Two datasets from roots of *Brassica napus* plants stressed with waterlogging for 12 h have been published already (Zou et al., 2013a,^2015^). In contrast to our study, the plants were grown in pots with sand and treated with waterlogging. Although this dataset is somewhat incomplete due to the missing reference genome at the time of publication, there is a good agreement with our dataset (Supplementary Figure 4 and Supplementary Table 2, columns AZ to BL). About 636 and 234 transcripts were hypoxia-responsive after 4 and 24 h in the present study and were also responsive to 6 and 12 h of waterlogging. The overlap with down-regulated genes was 222 and 89 transcripts for 4 and 24 h of hypoxia. The high number of genes only found in our dataset might be due to the different mapping basis, and probably not due to strong differences in expression (Supplementary Figure 4 compared with Supplementary Figure 5). Indeed, 45 and 42 HRG homologs were also induced by waterlogging of Zhongshuang 9 and GH01, respectively (Supplementary Table 2, column Q).

Another two rapeseed datasets have been obtained from about 2- to 3-day-old seedlings that were fully submerged for 12 or 6 h (Guo et al., 2020; Li et al., 2021), whose gene expression was based on the reference genome sequence Darmor (Chalhoub et al., 2014). Again, there was a good agreement of gene expression data in comparison with the expression data from roots from our dataset, despite of the different developmental stages and treatment conditions (Supplementary Table 2, columns BM to BT, Supplementary Table 7A). This similarity in gene expression was observed for up- and down-regulated genes and allowed for the definition of a *Brassica napus* hypoxic core response gene set (BnHRGs) containing 131 commonly up-regulated genes (Supplementary Table 7B) and 163 commonly down-regulated genes (Supplementary Table 7C).

### The Leaf Response Is Less Pronounced If Only Roots Are Affected by Hypoxia

In leaves, only a small number of genes was induced by root hypoxia, with little overlap between the two cultivars (Figure 1). The most enriched GO terms for Avatar were “2-(2′-methylthio)ethylmalate synthase activity” and “response to insect”, while for Zhongshuang 9 the categories “cation transmembrane transporter activity” (including zinc and iron) and “cellular response to heat” were enriched. Among the down-regulated genes, there was more functional overlap, including the categories “RNA methylation” and further ribosome-related categories as well as “cell wall” (Supplementary Table 3).

In leaves, there was no induction of HRGs (Supplementary Figure 2 and Supplementary Table 4) and no enrichment of GO categories associated with hypoxia (Supplementary Table 3). This does not come as a surprise, since the leaves were still in an aerobic atmosphere and with ongoing photosynthesis. The overlap with the starvation response of Arabidopsis (Usadel et al., 2008; Cookson et al., 2016), as previously observed in submerged rapeseed leaves (Wittig et al., 2021), was much lower in this dataset (Supplementary Table 4). There was also no indication for a modification of primary metabolism or photosynthesis due to root hypoxia (Supplementary Table 5), with the exception of one gene, PAD4, coding for an *ALANINE:GLYOXYLATE AMINOTRANSFERASE* (Parthasarathy et al., 2019), which was induced in leaves of the cultivar Avatar. At the 24-h-time point, there was no clear evidence for a drought response in leaves as a consequence of root damages under the stress (Supplementary Table 3). Besides the enhanced expression of the cytokinin signaling component *AHP4*, no hormone-related transcripts were differentially expressed in leaves of plants with hypoxic roots (Supplementary Table 6).

When we compared the new data to our previous analysis under submergence (Wittig et al., 2021), there was hardly any overlap between submerged leaves and leaves from plants with hypoxic roots (Supplementary Figure 6). Interestingly, the stressed roots after 24 h shared more induced or reduced transcripts with submerged leaves than the leaves from the two experiments (Supplementary Figure 6).

### Are There Differences in Gene Expression Between the Two Genotypes?

This analysis was done with two genotypes of *Brassica napus*, one European winter type (Avatar) and one Asian semi-winter type with a previously described high flooding tolerance (Zhongshuang 9, e.g., Zou et al., 2013b,^2014^). As mentioned above, the general response of roots to hypoxia was very similar between the two genotypes (Supplementary Table 2, columns R to AD, Supplementary Table 4 and Figure 1). There were only small differences at certain time points, and a statistical analysis using a complex comparison revealed no clear signatures (Supplementary Table 2, columns AR to AX). Also, the GO analysis of these complex comparisons did only show small differences, for example a stronger reduction of “xylosyltransferase activity” in Avatar (Supplementary Table 3). The observed differences might rather be due to differential expression of certain isoforms, while the overall transcript levels of a gene function were rather similar. Indeed, due to genome triplication and its tetraploid status (Nikolov and Tsiantis, 2017), rapeseed may contain up to 6 genes for a gene from Arabidopsis.

However, there were general differences in expression between both genotypes, independent of the stress treatment (Supplementary Table 2, columns AE to AQ, Supplementary Figure 7). These genotype-specific differences were similar to those observed before for submerged leaves (Wittig et al., 2021). In all root samples, about 2,900 transcripts were higher expressed in Avatar, while about 1,000 transcripts were higher in Zhongshuang 9. The difference in numbers might be partially caused by the difference in mapping rates between the two genotypes (Supplementary Table 1). A functional analysis classified the differentially expressed genes into certain categories, but there were only a few GOs enriched in our dataset (Supplementary Table 3). This suggests that gene copies, but not gene functions are differentially expressed between genotypes. Functional categories with higher expression levels in Avatar were enriched in ribosome function and translation, while in Zhongshuang 9 a few transport categories were enriched. Similar observations were made for leaves.

### Is There a Difference in Waterlogging Tolerance Between Both Cultivars?

**FIGURE 5:**
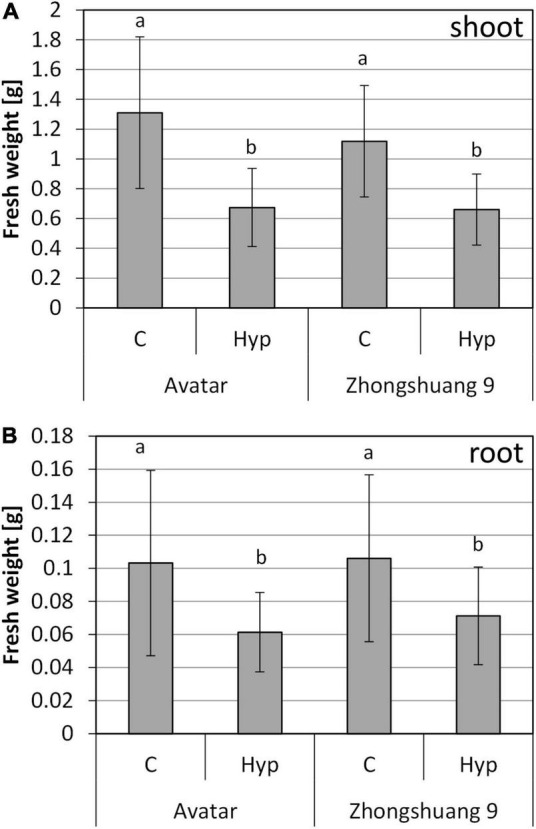
Fresh weight of shoots **(A)** and roots **(B)** of two *Brassica napus* cultivars (Avatar, Zhongshuang 9) in hydroponics after 7 days of root hypoxia, compared with aerated controls. At time of harvest, plants were 22 days old. Data are means ± SD of 4 experiments with 7-10 plants per experiment (*n* = 34 – 40). Different letters indicate significant differences (ANOVA and Tukey *post hoc* test, *p* < 0.05).

The transcriptional and biochemical analysis did not reveal obvious differences between the two rapeseed cultivars upon treatment with hypoxia in the root zone. We therefore asked whether differences exist in long-term response to hypoxia and waterlogging. First, we used the hydroponics system for a long-term gassing of plants with nitrogen in the root zone. After 7 days of treatment, plants were harvested, and fresh weight was determined (Figure 5). The treatment caused growth retardation of the plants, resulting in significantly lowered root and shoot fresh weights. Interestingly, there was also a slight decrease in the ratio of fresh weight to dry weight (Supplementary Figure 8A) indicating a slight drought due to root damages under prolonged root hypoxia. However, there were no differences between the two cultivars. Potentially, the treatment duration was too short, but the hydroponic system was not suitable for longer stress treatments. Furthermore, it might not perfectly mimic the situation in soil, or in agriculture.

**FIGURE 6:**
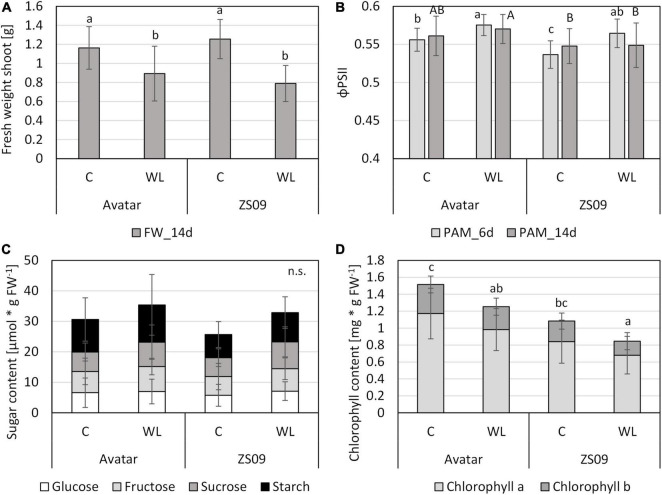
Performance of two *Brassica napus* cultivars (Avatar, Zhongshuang 9) on soil under waterlogging with tap water (“WL”) for up to 14 days. At time of harvest, plants were 29 days old. **(A)** Fresh weight of shoots after 14 days of stress treatment compared with controls. Data are means ± SD of 3 experiments with 10 plants per experiment (n = 30). **(B)** chlorophyll fluorescence (ΦPSII) after 6 and 14 days of stress treatment compared with controls. Data are means ± SD of 3 experiments with 10 plants per experiment (*n* = 30). **(C)** Content of carbohydrates [μmol * g FW^–1^] in leaves of after 14 days of stress. Data are means ± SD of 2 experiments with 6 plants per experiment (*n* = 12). **(D)** Chlorophyll content [mg * g FW^–1^] in leaves of after 14 days of stress. Data are means ± SD of 2 experiments with 8 plants per experiment (n = 16). Different letters indicate significant differences (ANOVA and Tukey *post hoc* test, *p* < 0.05). n.s., not significant.

Therefore, a more natural system was used, rapeseed plants grown on soil. After 15 days of growth on normoxic soil, plants were treated with waterlogging in the root zone. This treatment was done for 14 days. To evaluate fitness at an early timepoint in a non-invasive manner, a chlorophyll fluorescence parameter, the effective quantum yield of PSII (ΦPSII), was determined. This value slightly increased after 6 days of waterlogging in both genotypes (Figure 6B). Overall, the ΦPSII values for Zhongshuang 9 were slightly smaller than the values for Avatar, but the trend between control and stress treatment was the same. After 14 days of stress, the differences in ΦPSII between control and stress were no longer significant. The fresh weight in this experimental set-up significantly decreased under waterlogging, as shown in the hydroponic system (Figure 6A), but the changes were not as severe as expected. Again, no differences between the genotypes were found.

Two additional parameters were analyzed in this system, in order to reveal any effect of the modified stress system on the plant carbohydrate and pigment system. As shown for the short-term hydroponics system (Figure 4), the carbohydrate content of leaves from plants under waterlogging in soil did not respond to the stress treatment, even after 14 days of stress (Figure 6C). The chlorophyll content showed a tendency to decrease after stress treatment, but no significant differences between the two genotypes were observed (Figure 6D). These results suggest that waterlogging on soil in a controlled growth chamber might not be as severe as natural waterlogging in agriculture.

**FIGURE 7:**
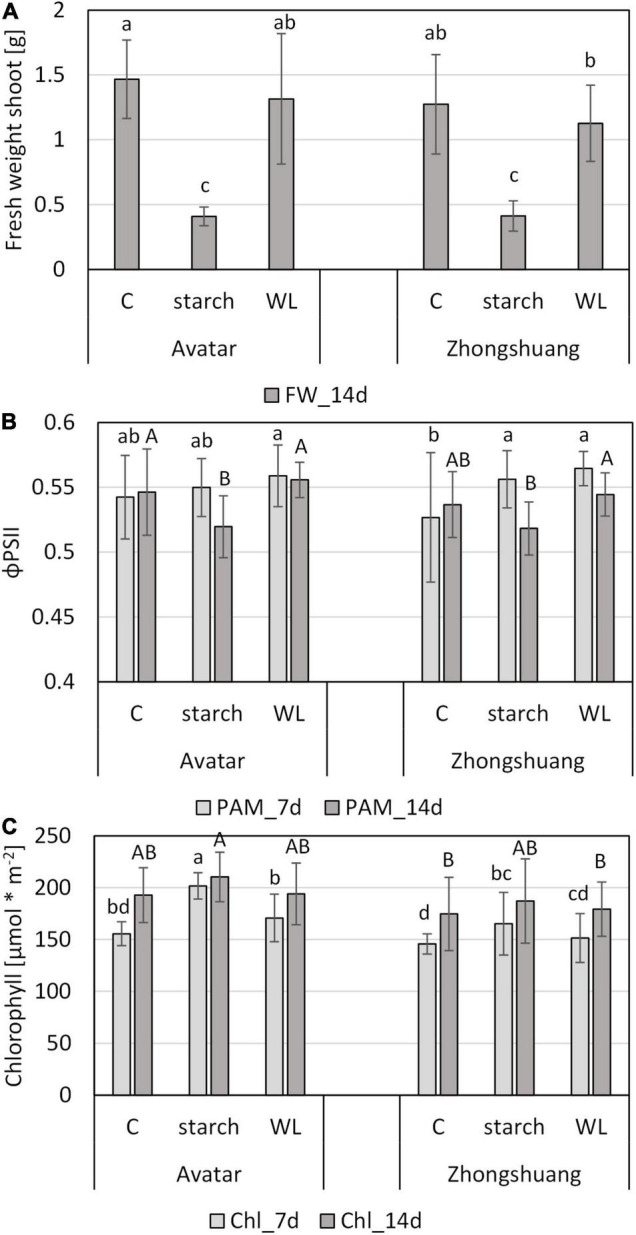
Performance of two *Brassica napus* cultivars (Avatar, Zhongshuang 9) on soil under waterlogging with a 0.1% starch solution (“starch”) or with distilled water (“WL”) for up to 14 days. At time of harvest, plants were 29 days old. **(A)** Fresh weight of shoots after 14 days of stress treatment compared with controls. **(B)** Chlorophyll fluorescence (ΦPSII) after 7 and 14 days of stress treatment compared with controls. **(C)** Chlorophyll content [μmol * m^–2^] in leaves of after 7 and 14 days of stress. Data are means ± SD of 3 experiments with 6 plants per experiment (*n* = 18). Different letters indicate significant differences (ANOVA and Tukey *post hoc* test, *p* < 0.05).

In order to establish a more natural system, we came across a waterlogging system with a starch solution that may cause a lower soil redox potential for plants grown in pots in climate chambers, which was successfully used for barley (Mano and Takeda, 2012; Miricescu et al., 2021). Strikingly, the waterlogging with a 0.1% starch solution strongly enhanced the severity of the stress treatment in comparison to waterlogging in distilled water (Figure 7 and Supplementary Figure 9) or in tap water (Figure 6). Here, the fresh weight of aboveground organs of stressed plants was reduced to 30% of the control plants, while in distilled water it was only reduced to 90% (Figure 7A), and in tap water to about 70% (Figure 6A). There were also indications for a stronger drought stress when waterlogging was done in starch solution since the ratio of fresh weight to dry weight was severely reduced (Supplementary Figure 8B). The analysis of chlorophyll fluorescence (ΦPSII) showed little changes after 7 days of stress, but a significant decrease after 14 days of waterlogging in a starch solution (Figure 7C). For chlorophyll, estimated here with a non-invasive method, we did not observe clear differences after 14 days of stress (Figure 7D).

In order to determine the causal reasons for these strong differences in plant growth, we also obtained chemical parameters of the waterlogging solutions in the two variants. While waterlogging with distilled water only resulted in mild decreases in oxygen content, starting at day 9, the oxygen content strongly decreased within the first 24 h of waterlogging in the starch solution, and remained at this low level for at least 10 days (Supplementary Figure 10A). Subsequently, the oxidation-reduction potential (ORP) decreased after 2 and 3 days of waterlogging with starch solution, but it remained relatively high in distilled water (Supplementary Figure 10B). The increase in both oxygen and ORP after 10 days of waterlogging might suggest a partial recovery of the system due to exhaustion of starch. The pH value temporarily decreased in the waterlogging treatment with starch (Supplementary Figure 10C), while the conductivity increased with time in both variants, with a slightly stronger increase in starch solution (Supplementary Figure 10D). The latter could be due to enhanced root death and subsequent release of ions into the solution.

## Discussion

### *Brassica napus* Roots Strongly Respond to Hypoxia

The oil crop plant *Brassica napus* is described to be very sensitive to soil waterlogging (Zou et al., 2014). It is not able to form aerenchyma in its roots as part of an avoidance strategy (Voesenek et al., 1999; Ploschuk et al., 2018), and will therefore experience hypoxia inside root tissues under waterlogging. We were therefore interested in its transcriptional response to this aspect of waterlogging stress to estimate whether rapeseed was able to induce biochemical acclimations as part of the tolerance strategy. After 4 and 24 h of root hypoxia, a high number of genes was differentially expressed in both cultivars compared to aerated controls (Supplementary Table 2, columns R to AD and Figure 1). More transcripts were down-regulated than up-regulated.

The functional analysis of up-regulated genes revealed the induction of a strong hypoxia response, as indicated by the enrichment of hypoxia-associated GO terms (Supplementary Table 3). Furthermore, there was a significant overlap with hypoxia-induced genes in Arabidopsis in general as well as with the hypoxia core response genes (HRGs, Mustroph et al., 2009; Figure 2 and Supplementary Figure 2, Supplementary Table 4). Among the hypoxia-induced genes were transcripts coding for fermentative enzymes (*ADH1*, *PDC1*, *PDC3*), glycolytic enzymes (*SUS1*, *FRK2*, *PFK3*, *PFK6*, *FBA6*) as well as transcriptional regulators (*HRE1*, *HRE2*, *HRA1*, *LBD41*). Both plant species are rather sensitive to waterlogging stress, and it remains to be evaluated whether differences in tolerance exist between the two species.

Glycolysis and fermentation are important processes to maintain energy balance under hypoxia since mitochondrial respiration is strongly inhibited. Their induction is therefore an essential component of the tolerance to low-oxygen stress. As Arabidopsis, also rapeseed was able to induce the expression of the associated genes under root hypoxia (Supplementary Table 2, columns R to AD), which may also result in higher enzyme activities as shown here for the ADH activity during prolonged stress treatment (Figure 3), and therefore might hint at an acclimation response of rapeseed plants. Mutants with a defect in fermentative enzymes indeed showed a lower tolerance to flooding-related stresses, for example in rice (Saika et al., 2006; Takahashi et al., 2014), maize (Schwartz, 1969; Johnson et al., 1994) or Arabidopsis (Jacobs et al., 1988; Ismond et al., 2003). Interestingly, the proposed enhancement of glycolysis and fermentation does not cause a carbohydrate limitation in our system (Figure 4), most likely due to ongoing photosynthesis in the leaves.

In addition, many plant species induced enzymes with alternative energy requirements upon hypoxia treatment, mainly sucrose synthase, the pyrophosphate-dependent phosphofructokinases (PFP) as well as PPDK (Huang et al., 2008; Mustroph et al., 2014, 2018; Atwell et al., 2015). Interestingly and unlike Arabidopsis (Mustroph et al., 2009), rapeseed induced two genes coding for the PFP alpha subunit in hypoxic roots, and this induction was stronger after 4 than after 24 h. This might be a hint for an alternative energy usage in *Brassica napus*. Several transcripts coding for PPDK and SUS are also among the hypoxia-induced genes in rapeseed (Supplementary Table 2, columns R to AD, Supplementary Table 5).

Among transcriptional regulators, the function of LBD41 is still unclear, but it is commonly induced by hypoxia in many plant species (Mustroph et al., 2010; Gasch et al., 2016). HRE1 and HRE2 are members of the group VII ERFs, which are important regulators of the hypoxia-specific transcription (Gibbs et al., 2011; Licausi et al., 2011). Both proteins are probably not involved in the initial response to hypoxia (Gasch et al., 2016), but most likely in the later stages of hypoxia (Licausi et al., 2010). HRA1 has been described as a negative regulator of hypoxia-specific transcription (Giuntoli et al., 2014, 2017). The transcriptional regulation of the hypoxic response in rapeseed might therefore be very similar to Arabidopsis.

Among the genes with reduced expression under hypoxia there are many whose products are involved in biosynthetic processes such as sterol, xylan and suberin biosynthesis, and in growth-related GO terms such as DNA replication, cell wall and cell proliferation (Supplementary Table 3). This is not surprising since it has been previously suggested that the hypoxia acclimation strategy includes down-regulation of growth and biosynthesis in Arabidopsis (Branco-Price et al., 2008; Mustroph et al., 2009). In accordance, growth of rapeseed plants is reduced under root hypoxia (Figure 5). Moreover, the low-oxygen quiescence strategy is a response of certain rice cultivars that show enhanced survival under relatively deep floods (Xu et al., 2006; Fukao and Bailey-Serres, 2008). Therefore, growth reduction of rapeseed plants could be a positive acclimation strategy under waterlogging (this work) as well as under submergence (Wittig et al., 2021).

Whether the down-regulation of growth and biosynthesis is an active process in *Brassica napus* remains to be elucidated. However, the fast down-regulation of many growth-related genes might suggest a controlled response. It is unlikely that the growth regulation is caused by sugar starvation since there is no indication for a carbohydrate starvation in rapeseed plants under root-zone hypoxia, and sugar content is rather higher than lower under stress (Figure 4). This phenomenon has been previously observed in waterlogged plants of many species, for example in wheat and maize (e.g., Huang and Johnson, 1995; Mustroph and Albrecht, 2003; Albrecht et al., 2004), tomato (Gharbi et al., 2009), potato (Biemelt et al., 1999), and rapeseed (Leul and Zhou, 1999). It might either be explained by a higher transport rate from the shoot, a reduced growth rate of roots, a limited sugar usage capacity in root cells, or a controlled down-regulation of root metabolism (Geigenberger et al., 2000; Albrecht et al., 2004; Gharbi et al., 2009).

The underlying factors for the observed growth reduction, however, are not known yet. Hormones known to play a role under flooding-related stresses are abscisic acid (ABA) and gibberellic acid (GA) (summarized in Bailey-Serres et al., 2012; Voesenek and Bailey-Serres, 2015). The GA-sensing DELLA proteins, who are responsible for growth reduction in rice under submergence (Fukao and Bailey-Serres, 2008), are not modified in their expression in rapeseed (Supplementary Table 2, columns R to AD; Supplementary Table 6). However, a transcript coding for the GA degradation enzyme *GIBBERELLIN 2-OXIDASE 3* (*GA2OX3*, Rieu et al., 2008) is strongly induced in hypoxic rapeseed roots (*BnaA05g09290D*), which might play a role in reduction of growth in this species, while its homolog *GA2OX2* is seldomly induced by hypoxia in Arabidopsis (Mustroph et al., 2010). An involvement of the stress hormone ABA in growth reduction is also possible since several genes coding for the biosynthesis enzyme 9-cis-epoxycarotenoid dioxygenase (*NCED*) are reduced in their expression (Supplementary Table 2, columns R to AD; Table 6). However, the HRG from Arabidopsis, *CYP707A3*, which is involved in the degradation of ABA (Okamoto et al., 2011), is not induced by hypoxia in rapeseed roots (Figure 2). In addition, other hormone pathways are differentially expressed and might be involved in the growth reduction, for example brassinosteroids, whose biosynthesis enzymes are strongly reduced in hypoxic roots (Supplementary Table 6).

The transcriptional response in this work was compared to other published data of rapeseed as well as to Arabidopsis. In two publications, young rapeseed seedlings (about 3 days old) were subjected to full submergence (Guo et al., 2020; Li et al., 2021). Despite the different systems, there was a substantial and significant overlap between the different treatments and developmental stages (Supplementary Table 2, columns BM to BT, Supplementary Table 7A). Many HRGs were among the induced genes under diverse flooding-related conditions. The direct comparison of our and earlier studies allowed us to define a set of hypoxia core response genes for *Brassica napus* (BnHRGs, Supplementary Tables 7B,C). 131 up-regulated and 163 down-regulated genes were in this dataset. While a large number of the up-regulated genes was also among HRGs in Arabidopsis (27), or was also hypoxia-responsive in Arabidopsis (90), a few genes seemed to be specific for rapeseed (14). Surprisingly, we found two transcription factors that are not yet described as HRGs in Arabidopsis, and that are rather linked to ABA, drought or wounding, namely *ETHYLENE RESPONSE FACTOR #111* (*ERF#111*, *ABR1*) and *DEHYDRATION-RESPONSIVE ELEMENT BINDING PROTEIN 2C* (*DREB2C*) (e.g., Lee et al., 2010; Kim et al., 2011; Bäumler et al., 2019). The importance of these transcription factors under waterlogging and hypoxia in rapeseed remains to be elucidated.

Comparing our expression data to the expression of Arabidopsis under hypoxia (e.g., Branco-Price et al., 2008; Mustroph et al., 2009, 2010 and references therein; Hsu et al., 2011) revealed a strong overlap (Supplementary Table 4). This included many, but not all HRGs. For example, the genes coding for *ACHT5*, *CYP707A3*, and *FHL* were not induced by hypoxia in *Brassica napus*. On the other hand, several genes were strongly induced in rapeseed by hypoxia, but not in Arabidopsis, for example *PEPTIDEMETHIONINE SULFOXIDE REDUCTASE 3* (*PMSR3*), ATP-dependent Clp protease (*AT1G33360*), *EXPANSIN-LIKE B1* (*EXLB1*), and two genes involved in hormone pathways already mentioned above (*AHP4*, *GA2OX3*). The function of these proteins in the context of hypoxic acclimation of rapeseed remains to be examined. A possible function of *PMSR3* could be the protection of proteins against oxidation of methionine residues (Sadanandom et al., 2000), but its induction in rapeseed seems to be only temporary.

There was also some overlap with genes commonly reduced by hypoxia in Arabidopsis. Although a previous work could not define a general set of down-regulated HRGs, there were seven genes that displayed a common root-specific down-regulation under hypoxia (Mustroph et al., 2009). Of those seven genes, three were also commonly reduced in hypoxic rapeseed roots, namely *CYP83B1*, *MYB DOMAIN PROTEIN 34* (*MYB34*) and *SLAC1 HOMOLOG 3* (*SLAH3*). The first two gene products are involved in tryptophan biosynthesis and its regulation, and thereby might affect glucosinolate biosynthesis (Celenza et al., 2005). Their common down-regulation supports our hypothesis that biosynthetic processes are mainly reduced under hypoxic conditions. The third gene codes for an anion channel, which recently has been described to be involved in depolarization of membranes due to cytoplasmic acidosis, for example caused by flooding and hypoxia (Lehmann et al., 2021). Indeed, Arabidopsis plants with a defect in *SLAH3* were more resistant to full submergence in darkness, and the down-regulation of its expression in Arabidopsis as well as rapeseed might be an advantage for survival.

Our experiments allow the conclusion that rapeseed roots can strongly respond to hypoxia in a coordinated way, and that this response is similar, but not identical to the response of Arabidopsis. Knowledge from the model plant *Arabidopsis thaliana*, for example about the transcriptional regulation of the hypoxia response and mechanisms of metabolic acclimation, might therefore be reasonably transferrable to the crop *Brassica napus*, in order to improve its tolerance against flooding. However, rapeseed has a much higher gene number due to whole-genome triplication and the tetraploid status (Nikolov and Tsiantis, 2017), making it a difficult species for genetic analyses. Indeed, most Arabidopsis genes have 2 to 8 isogenes in *Brassica napus*, which is also true for many HRGs. Most, but not all isogenes for one Arabidopsis HRG are regulated in a similar way in rapeseed (Supplementary Table 2, column Q; Supplementary Figure 2). It remains to be determined how the differential expression among isogenes is regulated, and whether previously defined promoter elements such as the HRPE (Gasch et al., 2016) are mutated or modified in some isogenes.

### *Brassica napus* Leaves Show Only a Minor Response to Root Hypoxia

Previously we have identified a set of genes that is strongly induced under submergence in leaves of two *Brassica napus* cultivars (Wittig et al., 2021). This analysis revealed a signature for carbohydrate starvation, which was in line with a severe decline in carbohydrate levels under water within a few hours, but hardly any induction of hypoxia-responsive genes. In the present work, leaves of *Brassica napus* plants with root hypoxia showed little changes in gene expression (Supplementary Table 2, columns R to AD) indicating that the shoot was not yet affected by the stress of the root system, including the carbohydrate levels (Figure 4). Previous analyses, however, have observed different responses in shoots of plants under root hypoxia or waterlogging. In Arabidopsis (Hsu et al., 2011) and cotton (Christianson et al., 2010), many transcripts in the shoot responded to the root stress after 12 and 24 h, respectively. An analysis of rapeseed under root waterlogging also revealed massive transcriptional changes in leaves after 36 and 72 h of stress (Lee et al., 2014). Only in poplar, a waterlogging treatment did not result in significant expression differences in leaves, as determined after 7 days of stress (Kreuzwieser et al., 2009). Possible explanations for such a small response in our system could be that (1) the timepoint was too early to observe transcriptional changes, (2) the stress treatment was not severe enough to induce changes in the shoot system, or (3) changes occurred mainly in other parts of the shoots, for example the meristem or the stem. Indeed, the work on Arabidopsis harvested whole shoots (Hsu et al., 2011), and this was also the case for the previous work on rapeseed (Lee et al., 2014).

Surprisingly, the low number of transcripts in leaves responding to root hypoxia are hardly related to any specific GO term, and almost no modifications were observed for photosynthesis-related genes, as it has been previously observed (Lee et al., 2014). Also, no modification of ROS metabolism and no induction of ABA biosynthesis genes that might indicate problems with water uptake were observed in our experiment. This is in accordance with the small changes within the physiological data, i.e., the sugar content in leaves (Figure 4), and no symptoms of wilting. Only after 7 days of stress treatment, the fresh weight in the hydroponic system was negatively affected (Figure 5), and a slightly lower water content was observed (Supplementary Figure 8A).

After longer durations of the stress in the soil waterlogging system, a decrease in chlorophyll content was detected, but only with the invasive method (Figjure 6D). Decreases in chlorophyll content in rapeseed under root waterlogging have been found before (Ashraf and Mehmood, 1990; Leul and Zhou, 1999; Lee et al., 2014), but they were more pronounced in the recovery phase. Surprisingly, the photosynthetic efficiency around PSII was only marginally affected under root waterlogging, as measured in the soil system (Figures 6B, 7C). These results suggest that photosynthesis was only mildly affected in our stress treatment, thus explaining little changes in gene expression or sugar status (Supplementary Table 2 and Figure 4). Therefore, a later timepoint might reveal more changes in gene expression in shoots, and potentially also a more severe treatment variant (Figure 7).

A common response of leaves in both genotypes was observed among down-regulated genes. Here, many genes associated with ribosomes and translation were lower expressed in leaves when roots were treated with hypoxia (Supplementary Table 3). This is a hint that there is indeed some regulation of gene expression in leaves of plants with roots under stress. The down-regulation of ribosomal proteins suggests a down-regulation of translation in general, as it has been observed before for Arabidopsis seedlings (Branco-Price et al., 2008; Mustroph et al., 2009). The signal that transmits the root stress to the shoots is, however, still unclear and remains to be solved.

### No Difference in the Response to Hypoxia and Waterlogging Between the Two Cultivars

One goal of this experimental set-up was to identify potential differences between two rapeseed cultivars, of which one had been described as tolerant toward waterlogging (e.g., Zou et al., 2013b,^2014^). However, in none of our experimental set-ups, root-zone hypoxia in hydroponics (Figures 3-5), waterlogging on soil (Figure 6), and waterlogging with a starch solution (Figure 7), we observed significant differences between the cultivars. Also, the gene expression analysis did not reveal clear differences in stress response between the genotypes (Supplementary Table 2, columns AR to AX). This might lead to the conclusion that the cultivars do not differ in their tolerance to waterlogging and root hypoxia, as previously demonstrated for treatment with submergence using the same two cultivars (Wittig et al., 2021). However, we cannot exclude a difference in tolerance at other developmental stages, in the recovery phase, or in field trials. It is also important to note that other rapeseed cultivars with contrasting response to waterlogging might exist. Furthermore, we have not evaluated whether differences in the genomic sequence of hypoxia-regulated genes exist between Avatar and Zhongshuang 9, and whether such differences might modify waterlogging and hypoxia tolerance. Indeed, certain single nucleotide polymorphisms (SNPs) that might be associated with submergence tolerance have been detected in a panel of rapeseed cultivars (Wang et al., 2020), but genome-wide association studies under waterlogging are still missing.

There are overall differences in gene expression between genotypes (Supplementary Figure 7 and Supplementary Table 2, columns AE to AQ), but they were seldomly related with a specific gene function (Supplementary Table 3). Ribosomal proteins are generally more expressed in Avatar than in Zhongshuang 9, and especially leaves show a slight enrichment of photosynthesis-associated genes, which might correlate with a slightly higher chlorophyll content (Figure 6D). Zhongshuang 9 showed even less functional categories with differential expression, which could be also due to a slightly lower mapping rate in comparison to the cultivar Avatar (Supplementary Table 1).

Previous analyses have suggested that Zhongshuang 9 is more tolerant to waterlogging than GH01 due to expression differences for several genes (Zou et al., 2013a,^2015^). However, the overall expression changes in response to the stress treatment in both cultivars were surprisingly similar (Supplementary Table 2, columns AZ to BL). In order to evaluate the previously observed expression differences, we extracted the respective *Brassica napus* gene IDs and looked for differential expression between our genotypes in the current genome assembly. However, none of the suggested candidates was differentially expressed between genotypes in our dataset (Supplementary Table 8A). Moreover, our overview reveals multiple genes coding for one gene function, and the sum over all transcripts might be more similar than single gene copies suggest, for example the nine transcripts coding for glyceraldehyde-3-phosphate dehydrogenase C subunit 1 (*GAPC1*, Supplementary Table 8A). In addition, a recent quantitative trait locus (QTL) analysis of the same two genotypes suggested another set of genes to be differentially expressed (Ding et al., 2020). However, there was no overlap of gene IDs between the two studies (Zou et al., 2015; Ding et al., 2020), and we could observe similar transcriptional changes for only a few transcripts in Zhongshuang 9 (Supplementary Table 8B). This suggests that (1) the candidate genes from both studies are not differentially expressed at all developmental stages or stress variants, (2) other genes might be responsible for the QTLs defined in Ding et al. (2020), or (3) the genotype Avatar does not differ as much from Zhongshuang 9 as GH01. In fact, most differences between the cultivars might occur only at the recovery phase after stress, as it has been recently demonstrated (Kuai et al., 2020a,b). Interestingly, the impact of nitrogen fertilizer on growth after flooding was as high or even higher than the impact of the genotype in these two publications.

More work is therefore needed, with the inclusion of more and diverse genotypes, in order to find and develop flooding-tolerant rapeseed genotypes. One improvement to previous waterlogging treatments could be the addition of 0.1% starch to the solution. The addition of starch strongly decreased the oxygen content and increased the reduction potential (Supplementary Figure 10), and therefore resembles more natural conditions than waterlogging with pure water, as previously demonstrated in barley (Mano and Takeda, 2012; Miricescu et al., 2021). In our hands, the stress treatment with a starch solution was more severe compared with waterlogging with tap water (Figure 7 and Supplementary Figure 9), and subsequent screening methods should consider this modified type of waterlogging stress, together with the addition of a recovery phase.

## Conclusion

This work demonstrates that rapeseed is indeed able to strongly respond to waterlogging-associated hypoxia in the root zone at the transcriptional level. These responses might indicate an acclimation response to the stress, but they could also include responses to stress damage. A core hypoxia response for rapeseed plants could be defined, which can be used for future studies. However, a genotype-specific response to hypoxia and waterlogging between cultivars from different origin (Europe and Asia) could not be detected. However, we cannot exclude that SNPs related to tolerance are more important than overall transcriptional levels. In future, differences in gene sequences should be analyzed as well, for example by using a genome-wide association mapping approach, potentially together with QTL analyses. So far, only very few studies on rapeseed under flooding stress contain sufficient data for such an approach (Ding et al., 2020; Wang et al., 2020), and more work is required in this direction. The present dataset and a modified waterlogging treatment of plants grown in pots by use of a starch solution might help to identify potential tolerance-related genes in certain QTL regions, which ultimately could result in waterlogging-tolerant rapeseed genotypes.

## Data Availability Statement

The datasets presented in this study can be found in online repositories. The names of the repository/repositories and accession number(s) can be found below: National Center for Biotechnology Information (NCBI) BioProject database under accession number GSE180262.

## Author Contributions

AM designed the experiments, analyzed the data and wrote the manuscripts. BB developed the methods. SA, MK, PW, and AM performed the experiments. All authors contributed to the article and approved the submitted version.

## Conflict of Interest

The authors declare that the research was conducted in the absence of any commercial or financial relationships that could be construed as a potential conflict of interest.

## Publisher’s Note

All claims expressed in this article are solely those of the authors and do not necessarily represent those of their affiliated organizations, or those of the publisher, the editors and the reviewers. Any product that may be evaluated in this article, or claim that may be made by its manufacturer, is not guaranteed or endorsed by the publisher.

## Abbreviations

ADH: alcohol dehydrogenase
ERF: ethylene response factor
FDR: false discovery rate
GO: gene ontology
HRG: hypoxia core-response gene
PDC: pyruvate decarboxylase
SNP: single nucleotide polymorphism
QTL: quantitative trail locus.

## References

[B1] Albrecht G. Mustroph A. Fox T. C. (2004). Sugar and fructan accumulation during metabolic adjustment between respiration and fermentation under low oxygen conditions in wheat roots. *Physiol. Plant.* 120 93–105. 10.1111/j.0031-9317.2004.0205.x 15032881

[B2] Arnon D. xI. (1949). Copper enzymes in isolated chloroplasts. Polyphenoloxidase in *Beta vulgaris*. *Plant Physiol.* 24 1–15. 10.1104/pp.24.1.1 16654194 PMC437905

[B3] Ashraf M. Mehmood S. (1990). Effects of waterlogging on growth and some physiological parameters of four *Brassica* species. *Plant Soil* 121 203–209. 10.1007/bf00012313

[B4] Atwell B. J. Greenway H. Colmer T. D. (2015). Efficient use of energy in anoxia-tolerant plants with focus on germinating rice seedlings. *New Phytol.* 206 36–56. 10.1111/nph.13173 25472708

[B5] Bailey-Serres J. Fukao T. Gibbs D. J. Holdsworth M. J. Lee S. C. Licausi F. (2012). Making sense of low oxygen sensing. *Trends Plant Sci.* 17 129–138. 10.1016/j.tplants.2011.12.004 22280796

[B6] Bäumler J. Riber W. Klecker M. Müller L. Dissmeyer N. Weig A. R. (2019). AtERF#111/ABR1 is a transcriptional activator involved in the wounding response. *Plant J.* 100 969–990. 31385625 10.1111/tpj.14490

[B7] Biemelt S. Hajirezaei M. R. Melzer M. Albrecht G. Sonnewald U. (1999). Sucrose synthase activity does not restrict glycolysis in roots of transgenic potato plants under hypoxic conditions. *Planta* 210 41–49. 10.1007/s004250050652 10592031

[B8] Blöschl G. Hall J. Viglione A. Perdigão R. A. P. Parajka J. Merz B. (2019). Changing climate both increases and decreases European river floods. *Nature* 573 108–111. 10.1038/s41586-019-1495-6 31462777

[B9] Bradford M. M. (1976). A rapid and sensitive method for the quantitation of microgram quantities of protein utilizing the principle of protein-dye binding. *Anal. Biochem.* 72 248–254. 10.1006/abio.1976.9999 942051

[B10] Branco-Price C. Kaiser K. A. Jang C. J. Larive C. K. Bailey-Serres J. (2008). Selective mRNA translation coordinates energetic and metabolic adjustments to cellular oxygen deprivation and reoxygenation in *Arabidopsis thaliana*. *Plant J.* 56 743–755. 10.1111/j.1365-313X.2008.03642.x 18665916

[B11] Bray N. L. Pimentel H. Melsted P. Pachter L. (2016). Near-optimal probabilistic RNA-seq quantification. *Nat. Biotechnol.* 34 525–527. 10.1038/nbt.3519 27043002

[B12] Celenza J. L. Quiel J. A. Smolen G. A. Merrikh H. Silvestro A. R. Normanly J. (2005). The *Arabidopsis* ATR1 Myb transcription factor controls indolic glucosinolate homeostasis. *Plant Physiol.* 137 253–262. 10.1104/pp.104.054395 15579661 PMC548856

[B13] Chalhoub B. Denoeud F. Liu S. Parkin I. A. Tang H. Wang X. (2014). Early allopolyploid evolution in the post-Neolithic *Brassica napus* oilseed genome. *Science* 345 950–953. 10.1126/science.1253435 25146293

[B14] Christianson J. A. Llewellyn D. J. Dennis E. S. Wilson I. W. (2010). Global gene expression responses to waterlogging in roots and leaves of cotton (*Gossypium hirsutum* L.). *Plant Cell Physiol.* 51 21–37. 10.1093/pcp/pcp163 19923201

[B15] Cookson S. J. Yadav U. P. Klie S. Morcuende R. Usadel B. Lunn J. E. (2016). Temporal kinetics of the transcriptional response to carbon depletion and sucrose readdition in *Arabidopsis* seedlings. *Plant Cell Environ.* 39 768–786. 10.1111/pce.12642 26386165

[B16] Ding X. Y. Xu J. S. Huang H. Qiao X. Shen M. Z. Cheng Y. (2020). Unraveling waterlogging tolerance-related traits with QTL analysis in reciprocal intervarietal introgression lines using genotyping by sequencing in rapeseed (*Brassica napus* L). *J. Integr. Agric.* 19 2–11.

[B17] Fukao T. Bailey-Serres J. (2008). Submergence tolerance conferred by Sub1A is mediated by SLR1 and SLRL1 restriction of gibberellin responses in rice. *Proc. Natl. Acad. Sci. U.S.A.* 105 16814–16819. 10.1073/pnas.0807821105 18936491 PMC2575502

[B18] Gasch P. Fundinger M. Müller J. T. Lee T. Bailey-Serres J. Mustroph A. (2016). Redundant ERF-VII transcription factors bind to an evolutionarily conserved cis-motif to regulate hypoxia-responsive gene expression in *Arabidopsis*. *Plant Cell* 28 160–180. 10.1105/tpc.15.00866 26668304 PMC4746684

[B19] Geigenberger P. Fernie A. R. Gibon Y. Christ M. Stitt M. (2000). Metabolic activity decreases as an adaptive response to low internal oxygen in growing potato tubers. *Biol. Chem.* 381 723–740. 10.1515/BC.2000.093 11030430

[B20] Gharbi I. Ricard B. Smiti S. Bizid E. Brouquisse R. (2009). Increased hexose transport in the roots of tomato plants submitted to prolonged hypoxia. *Planta* 230 441–448. 10.1007/s00425-009-0941-3 19437034

[B21] Gibbs D. J. Lee S. C. Isa N. M. Gramuglia S. Fukao T. Bassel G. W. (2011). Homeostatic response to hypoxia is regulated by the N-end rule pathway in plants. *Nature* 479 415–418. 10.1038/nature10534 22020279 PMC3223408

[B22] Giuntoli B. Lee S. C. Licausi F. Kosmacz M. Oosumi T. van Dongen J. T. (2014). A trihelix DNA binding protein counterbalances hypoxia-responsive transcriptional activation in *Arabidopsis*. *PLoS Biol.* 12:e1001950. 10.1371/journal.pbio.1001950 25226037 PMC4165759

[B23] Giuntoli B. Licausi F. van Veen H. Perata P. (2017). Functional balancing of the hypoxia regulators RAP2.12 and HRA1 takes place in vivo in *Arabidopsis thaliana* plants. *Front. Plant Sci.* 8:591. 10.3389/fpls.2017.00591 28487707 PMC5403939

[B24] Guo Y. Chen J. Kuang L. Wang N. Zhang G. Jiang L. (2020). Effects of waterlogging stress on early seedling development and transcriptomic responses in *Brassica napus*. *Mol. Breed.* 40:85.

[B25] Hsu F. C. Chou M. Y. Peng H. P. Chou S. J. Shih M. C. (2011). Insights into hypoxic systemic responses based on analyses of transcriptional regulation in *Arabidopsis*. *PLoS One* 6:e28888. 10.1371/journal.pone.0028888 22194941 PMC3240646

[B26] Huang B. Johnson J. (1995). Root respiration and carbohydrate status of two wheat genotypes in response to hypoxia. *Ann. Bot.* 75 427–432. 10.1006/anbo.1995.1041

[B27] Huang S. Colmer T. D. Millar A. H. (2008). Does anoxia tolerance involve altering the energy currency towards PPi? *Trends Plant Sci.* 13 221–227. 10.1016/j.tplants.2008.02.007 18439868

[B28] Ismond K. P. Dolferus R. de Pauw M. Dennis E. S. Good A. G. (2003). Enhanced low oxygen survival in *Arabidopsis* through increased metabolic flux in the fermentative pathway. *Plant Physiol.* 132 1292–1302. 10.1104/pp.103.022244 12857811 PMC167069

[B29] Jacobs M. Dolferus R. Van den Bossche D. (1988). Isolation and biochemical analysis of ethyl methanesulfonate-induced alcohol dehydrogenase null mutants of *Arabidopsis thaliana* (L.) Heynh. *Biochem. Genet.* 26 105–122. 10.1007/BF00555492 3377754

[B30] Johnson J. R. Cobb B. G. Drew M. C. (1994). Hypoxic induction of anoxia tolerance in roots of Adh1 null *Zea mays* L. *Plant Physiol.* 105 61–67. 10.1104/pp.105.1.61 12232186 PMC159329

[B31] Kim J. S. Mizoi J. Yoshida T. Fujita Y. Nakajima J. Ohori T. (2011). An ABRE promoter sequence is involved in osmotic stress-responsive expression of the DREB2A gene, which encodes a transcription factor regulating drought-inducible genes in *Arabidopsis*. *Plant Cell Physiol.* 52 2136–2146. 10.1093/pcp/pcr143 22025559

[B32] Kreuzwieser J. Hauberg J. Howell K. A. Carroll A. Rennenberg H. Millar A. H. (2009). Differential response of gray poplar leaves and roots underpins stress adaptation during hypoxia. *Plant Physiol.* 149 461–473. 10.1104/pp.108.125989 19005089 PMC2613732

[B33] Kuai J. Li X. Li Z. Xie Y. Wang B. Zhou G. (2020a). Leaf carbohydrates assimilation and metabolism affect seed yield of rapeseed with different waterlogging tolerance under the interactive effects of nitrogen and waterlogging. *J. Agron. Crop Sci.* 206 823–836. 10.1111/jac.12430

[B34] Kuai J. Li X. Xie Y. Li Z. Wang B. Zhou G. (2020b). Leaf characteristics at recovery stage affect seed oil and protein content under the interactive effects of nitrogen and waterlogging in rapeseed. *Agriculture* 10:207. 10.3390/agriculture10060207

[B35] Kundzewicz Z.W. Kanae S. Seneviratne S.I. Handmer J. Nicholls N. Peduzzi P. (2014). Flood risk and climate change: global and regional perspectives. *Hydrol. Sci. J.* 59 1–28. 10.1163/9789004447615_002

[B36] Lee S. J. Kang J. Y. Park H. J. Kim M. D. Bae M. S. Choi H. I. (2010). DREB2C interacts with ABF2, a bZIP protein regulating abscisic acid-responsive gene expression, and its overexpression affects abscisic acid sensitivity. *Plant Physiol.* 153 716–727. 10.1104/pp.110.154617 20395451 PMC2879808

[B37] Lee T. A. Bailey-Serres J. (2019). Integrative analysis from the epigenome to translatome uncovers patterns of dominant nuclear regulation during transient stress. *Plant Cell* 31 2573–2595. 10.1105/tpc.19.00463 31519798 PMC6881120

[B38] Lee Y. H. Kim K. S. Jang Y. S. Hwang J. H. Lee D. H. Choi I. C. H. (2014). Global gene expression responses to waterlogging in leaves of rape seedlings. *Plant Cell Rep.* 33 289–299. 10.1007/s00299-013-1529-8 24384821

[B39] Lehmann J. Jørgensen M. E. Fratz S. Müller H. M. Kusch J. Scherzer S. (2021). Acidosis-induced activation of anion channel SLAH3 in the flooding-related stress response of *Arabidopsis*. *Curr. Biol.* 31 3575–3585.e9. 10.1016/j.cub.2021.06.018 34233161

[B40] Leul M. Zhou W. J. (1999). Alleviation of waterlogging damage in winter rape by uniconazole application: effects on enzyme activity, lipid peroxidation, and membrane integrity. *J. Plant Growth Regul.* 18 9–14. 10.1007/pl00007046 10467014

[B41] Li J. Iqbal S. Zhang Y. Chen Y. Tan Z. Ali U. (2021). Transcriptome analysis reveals genes of flooding-tolerant and flooding-sensitive rapeseeds differentially respond to flooding at the germination stage. *Plants (Basel)* 10 693. 10.3390/plants10040693 33916802 PMC8065761

[B42] Licausi F. Kosmacz M. Weits D. A. Giuntoli B. Giorgi F. M. Voesenek L. A. C. J. (2011). Oxygen sensing in plants is mediated by an N-end rule pathway for protein destabilization. *Nature* 479 419–422. 10.1038/nature10536 22020282

[B43] Licausi F. van Dongen J. T. Giuntoli B. Novi G. Santaniello A. Geigenberger P. (2010). HRE1 and HRE2, two hypoxia-inducible ethylene response factors, affect anaerobic responses in *Arabidopsis thaliana*. *Plant J.* 62 302–315. 10.1111/j.1365-313X.2010.04149.x 20113439

[B44] Mano Y. Takeda K. (2012). Accurate evaluation and verification of varietal ranking for flooding tolerance at the seedling stage in barley (*Hordeum vulgare* L.). *Breed. Sci.* 62 3–10. 10.1270/jsbbs.62.3 23136508 PMC3405954

[B45] McCarthy D. J. Chen Y. Smyth G. K. (2012). Differential expression analysis of multifactor RNA-Seq experiments with respect to biological variation. *Nucleic Acids Res.* 40 4288–4297. 10.1093/nar/gks042 22287627 PMC3378882

[B46] Miricescu A. Byrne T. Doorly C. M. Ng C. K. Y. Barth S. Graciet E. (2021). Experimental comparison of two methods to study barley responses to partial submergence. *Plant Methods* 17 40. 10.1186/s13007-021-00742-5 33849604 PMC8045378

[B47] Müller J. T. van Veen H. Bartylla M. M. Akman M. Pedersen O. Sun P. (2021). Keeping the shoot above water - submergence triggers antithetical growth responses in stems and petioles of watercress (*Nasturtium officinale*). *New Phytol.* 229 140–155. 10.1111/nph.16350 31792981

[B48] Mustroph A. (2018). Improving flooding tolerance of crop plants. *Agronomy (Basel)* 8:160. 10.1093/aobpla/plu016 24876298 PMC4011469

[B49] Mustroph A. Albrecht G. (2003). Tolerance of crop plants to oxygen deficiency stress: fermentative activity and photosynthetic capacity of entire seedlings under hypoxia and anoxia. *Physiol. Plant.* 117 508–520. 10.1034/j.1399-3054.2003.00051.x 12675741

[B50] Mustroph A. Boamfa E. I. Laarhoven L. J. Harren F. J. Pörs Y. Grimm B. (2006). Organ specific analysis of the anaerobic primary metabolism in rice and wheat seedlings II: light exposure reduces needs for fermentation and extends survival during anaerobiosis. *Planta* 225 139–152. 10.1007/s00425-006-0336-7 16802177

[B51] Mustroph A. Hess N. Sasidharan R. (2014). “Hypoxic energy metabolism and PPi as an alternative energy currency,” in *Low-Oxygen Stress in Plants, Plant Cell Monographs*, Vol. 21 eds van Dongen J. T. Licausi F. (Wien: Springer-Verlag), 165–184. 10.1016/j.plantsci.2020.110572

[B52] Mustroph A. Lee S. C. Oosumi T. Zanetti M. E. Yang H. Ma K. (2010). Cross-kingdom comparison of transcriptomic adjustments to low-oxygen stress highlights conserved and plant-specific responses. *Plant Physiol.* 152 1484–1500. 10.1104/pp.109.151845 20097791 PMC2832244

[B53] Mustroph A. Steffens B. Sasidharan R. (2018). Signalling interactions in flooding tolerance. *Ann. Plant Rev. Online* 1 1–42.

[B54] Mustroph A. Zanetti M. E. Jang C. J. Holtan H. E. Repetti P. P. Galbraith D. W. (2009). Profiling translatomes of discrete cell populations resolves altered cellular priorities during hypoxia in *Arabidopsis*. *Proc. Natl. Acad. Sci. U.S.A.* 106 18843–18848. 10.1073/pnas.0906131106 19843695 PMC2764735

[B55] Nikolov L. A. Tsiantis M. (2017). Using mustard genomes to explore the genetic basis of evolutionary change. *Curr. Opin. Plant Biol.* 36 119–128. 10.1016/j.pbi.2017.02.005 28285128

[B56] Okamoto M. Kushiro T. Jikumaru Y. Abrams S. R. Kamiya Y. Seki M. (2011). ABA 9’-hydroxylation is catalyzed by CYP707A in *Arabidopsis*. *Phytochemistry* 72 717–722. 10.1016/j.phytochem.2011.02.004 21414645

[B57] Park B. S. Yao T. Seo J. S. Wong E. C. C. Mitsuda N. Huang C. H. (2018). *Arabidopsis* NITROGEN LIMITATION ADAPTATION regulates ORE1 homeostasis during senescence induced by nitrogen deficiency. *Nat. Plants* 4 898–903. 10.1038/s41477-018-0269-8 30374089

[B58] Parthasarathy A. Adams L. E. Savka F. C. Hudson A. O. (2019). The *Arabidopsis thaliana* gene annotated by the locus tag At3g08860 encodes alanine aminotransferase. *Plant Direct* 3:e00171. 10.1002/pld3.171 31549019 PMC6750192

[B59] Pekel J. F. Cottam A. Gorelick N. Belward A. S. (2016). High-resolution mapping of global surface water and its long-term changes. *Nature* 540 418–422. 10.1038/nature20584 27926733

[B60] Ploschuk R. A. Miralles D. J. Colmer T. D. Ploschuk E. L. Striker G. G. (2018). Waterlogging of winter crops at early and late stages: impacts on leaf physiology, growth and yield. *Front. Plant Sci.* 9:1863. 10.3389/fpls.2018.01863 PMC6306497 30619425

[B61] Rieu I. Eriksson S. Powers S. J. Gong F. Griffiths J. Woolley L. (2008). Genetic analysis reveals that C_19_-GA 2-oxidation is a major gibberellin inactivation pathway in *Arabidopsis*. *Plant Cell* 20 2420–2436. 10.1105/tpc.108.058818 18805991 PMC2570722

[B62] Sadanandom A. Poghosyan Z. Fairbairn D. J. Murphy D. J. (2000). Differential regulation of plastidial and cytosolic isoforms of peptide methionine sulfoxide reductase in *Arabidopsis*. *Plant Physiol.* 123 255–264. 10.1104/pp.123.1.255 10806242 PMC58999

[B63] Saika H. Matsumura H. Takano T. Tsutsumi N. Nakazono M. (2006). A point mutation of Adh1 gene is involved in the repression of coleoptile elongation under submergence in rice. *Breed. Sci.* 56 69–74. 10.1270/jsbbs.56.69 26081539

[B64] Sasidharan R. Bailey-Serres J. Ashikari M. Atwell B. J. Colmer T. D. Fagerstedt K. (2017). Community recommendations on terminology and procedures used in flooding and low oxygen stress research. *New Phytol.* 214 1403–1407. 10.1111/nph.14519 28277605

[B65] Schwartz D. (1969). An example of gene fixation resulting from selective advantage in suboptimal conditions. *Am. Nat.* 103 479–481. 10.1086/282615

[B66] Takahashi H. Greenway H. Matsumura H. Tsutsumi N. Nakazono M. (2014). Rice alcohol dehydrogenase 1 promotes survival and has a major impact on carbohydrate metabolism in the embryo and endosperm when seeds are germinated in partially oxygenated water. *Ann. Bot.* 113 851–859. 10.1093/aob/mct305 24431339 PMC3962239

[B67] Trenberth K. E. Dai A. Van Der Schrier G. Jones P. D. Barichivich J. Briffa K. R. (2014). Global warming and changes in drought. *Nat. Clim. Chang.* 4 17–22.

[B68] Usadel B. Bläsing O. E. Gibon Y. Retzlaff K. Höhne M. Günther M. (2008). Global transcript levels respond to small changes of the carbon status during progressive exhaustion of carbohydrates in *Arabidopsis* rosettes. *Plant Physiol.* 146 1834–1861. 10.1104/pp.107.115592 18305208 PMC2287354

[B69] Voesenek L. A. C. J. Bailey-Serres J. (2015). Flood adaptive traits and processes: an overview. *New Phytol.* 206 57–73. 10.1111/nph.13209 25580769

[B70] Voesenek L. A. C. J. Armstrong W. Bogemann G. M. Colmer T. D. (1999). A lack of aerenchyma and high rates of radial oxygen loss from the root base contribute to waterlogging intolerance in *Brassica napus*. *Aust. J. Plant Physiol.* 26 87–93. 10.1071/pp98086

[B71] Wang X. Sun L. Li W. Peng M. Chen F. Zhang W. (2020). Dissecting the genetic mechanisms of waterlogging tolerance in *Brassica napus* through linkage mapping and a genome-wide association study. *Ind. Crops Prod.* 147:112269. 10.1016/j.indcrop.2020.112269

[B72] Waters I. Morell S. Greenway H. Colmer D. (1991). Effects of anoxia on wheat seedlings. II influence of O_2_ supply prior to anoxia on tolerance to anoxia, alcoholic fermentation, and sugar levels. *J. Exp. Bot.* 42 1437–1447. 10.1093/jxb/42.11.1437 12432039

[B73] Wittig P. R. Ambros S. Müller J. T. Bammer B. Álvarez-Cansino L. Konnerup D. (2021). Two *Brassica napus* cultivars differ in gene expression, but not in their response to submergence. *Physiol. Plant.* 171 400–415. 10.1111/ppl.13251 33099772

[B74] Wollmer A. -C. Pitann B. Mühling K. H. (2018). Waterlogging events during stem elongation or flowering affect yield of oilseed rape (*Brassica napus* L.) but not seed quality. *J. Agron. Crop Sci.* 204 165–174. 10.1111/jac.12244

[B75] Xu J. Qiao X. Tian Z. Zhang X. Zou X. Cheng Y. (2018). Proteomic analysis of rapeseed root response to waterlogging stress. *Plants (Basel)* 7:E71. 10.3390/plants7030071 30205432 PMC6160990

[B76] Xu K. Xu X. Fukao T. Canlas P. Maghirang-Rodriguez R. Heuer S. (2006). Sub1A is an ethylene-response-factor-like gene that confers submergence tolerance to rice. *Nature* 442 705–708. 10.1038/nature04920 16900200

[B77] Yamauchi T. Colmer T. D. Pedersen O. Nakazono M. (2018). Regulation of root traits for internal aeration and tolerance to soil waterlogging-flooding stress. *Plant Physiol.* 176 1118–1130. 10.1104/pp.17.01157 29118247 PMC5812745

[B78] Zou X. L. Tan X. Y. Hu C. W. Zeng L. Lu G. Y. Fu G. P. (2013a). The transcriptome of *Brassica napus* L. roots under waterlogging at the seedling stage. *Intern. J. Mol. Sci.* 14 2637–2651. 10.3390/ijms14022637 23358252 PMC3588007

[B79] Zou X. L. Cong Y. Cheng Y. Lu G. Y. Zhang X. K. (2013b). “Screening and identification of waterlogging tolerant rapeseed (*Brassica napus* L.) during germination stage,” in *Proceedings of the 3rd International Conference on Intelligent System Design and Engineering Applications (ISDEA)*, Hong Kong, 1248–1253.

[B80] Zou X. L. Hu C. W. Zeng L. Cheng Y. Xu M. Y. Zhang X. K. (2014). A comparison of screening methods to identify waterlogging tolerance in the field in *Brassica napus* L. during plant ontogeny. *PLoS One* 9:e89731. 10.1371/journal.pone.0089731 24594687 PMC3940661

[B81] Zou X. L. Zeng L. Lu G. Y. Cheng Y. Xu J. S. Zhang X. K. (2015). Comparison of transcriptomes undergoing waterlogging at the seedling stage between tolerant and sensitive varieties of *Brassica napus* L. *J. Integr. Agric.* 14 1723–1734. 10.1016/s2095-3119(15)61138-8

