# Supplementary material for: Transcriptional Response of Two Brassica napus Cultivars to Short-Term Hypoxia in the Root Zone
Source: Front Plant Sci. 2022 Apr 29;13:897673. doi: 10.3389/fpls.2022.897673 (PMC9100894; doi:10.3389/fpls.2022.897673)
Supplement: Supplementary file 1 [file Data_Sheet_1.DOCX]

Supplementary Material

# Supplementary Figures

**Figure S1:** Venn diagrams showing overlaps between treatments of induced (A,B) or repressed (C,D) genes of two *Brassica napus* cultivars (Avatar, Zhongshuang 9) in hydroponics after 4 and 24 h of root hypoxia, compared with aerated controls. **(A,C)** Common response in Avatar; **(B,D)** Common response in Zhongshuang 9. R, root; L, leaf.

**Figure S2:** Heatmap of hypoxia core-response genes (Mustroph et al. 2009) from the *Brassica napus* expression data. Values are signal-log ratios (SLR) of root-zone hypoxia vs. aerated control. AV, Avatar; ZS, Zhongshuang 9. The color intensity reflects the SLR values (blue, -3; yellow, +3). Crossed cells represent values that are not significant (FDR > 0.01). All expressed *B. napus* transcripts for each Arabidopsis gene are shown.

 **Figure S2,** continued

**Figure S3:** ADH activity [nmol * mg prot^-1^ * min^-1^] in roots of two *Brassica napus* cultivars (Avatar - blue, Zhongshuang 9 - red) in hydroponics after 4 and 24 hours of root hypoxia (Hyp), compared with aerated controls (C). At the time of the treatment start, plants were 15 days old. Values of 4 to 6 replicates are presented in box plots with medians and first and third quartile (http://shiny.chemgrid.org/boxplotr/). Single activity values are shown with circles. n.s., no significant differences were found between the two genotypes at any time point (ANOVA, P < 0.05).

**Figure S4:** Comparison of the hypoxic response in this work with two other publications (Zou et al., 2013a; Zou et al., 2015). Venn diagrams show overlaps between treatments of induced **(A,B)** or repressed **(C,D)** genes after 4 h (A,C) and 24 h (B,D) of root hypoxia, compared with aerated controls. Data for 12 h of Zhongshuang 9 are from Zou et al. (2013a), data for 12 h of GH01 are from Zou et al. (2015). R, root.

**Figure S5:** Comparison of the hypoxic response in this work (filtered genes set) with two other publications (Zou et al., 2013a; Zou et al., 2015). Venn diagrams show overlaps between treatments of induced **(A,B)** or repressed **(C,D)** genes after 4 h (A,C) and 24 h (B,D) of root hypoxia, compared with aerated controls. In contrast to Fig. S2, only transcripts with expression data for all three datasets were considered. Data for 12 h of Zhongshuang 9 are from Zou et al. (2013a), data for 12 h of GH01 are from Zou et al. (2015). R, root.

**Figure S6:** Venn diagrams showing overlaps between treatments in this study and in our earlier study on full submergence (sub, Wittig et al., 2021, labelled with green color). Venn diagrams show overlaps between treatments of induced **(A,B)** or repressed **(C,D)** genes. (A,C) Common response in Avatar; (B,D) Common response in Zhongshuang 9. R, root; L, leaf.

**Figure S7:** Venn diagrams showing overlaps of genotype-specific genes in a direct comparison between the two *Brassica napus* cultivars Avatar and Zhongshuang 9. Data from our earlier study on submergence were also included here (Wittig et al., 2021, labelled with green color). **(A)** Genes with higher expression level in Avatar compared with Zhongshuang 9 in root samples; **(B)** Genes with higher expression level in Zhongshuang 9 compared with Avatar in root samples; **(C)** Genes with higher expression level in Avatar compared with Zhongshuang 9 in leaf samples; **(D)** Genes with higher expression level in Zhongshuang 9 compared with Avatar in leaf samples. Numbers with red font in C and D mark numbers of genes of unstressed leaf samples and genotype-specific genes under stress treatment, respectively. C, control; Hyp, root hypoxia; SUB, full submergence.

**Figure S8:** Ratio of Fresh weight (FW) to dry weight (DW) from experiments shown in **(A)** Fig. 4A and **(B)** Fig. 6A.

**
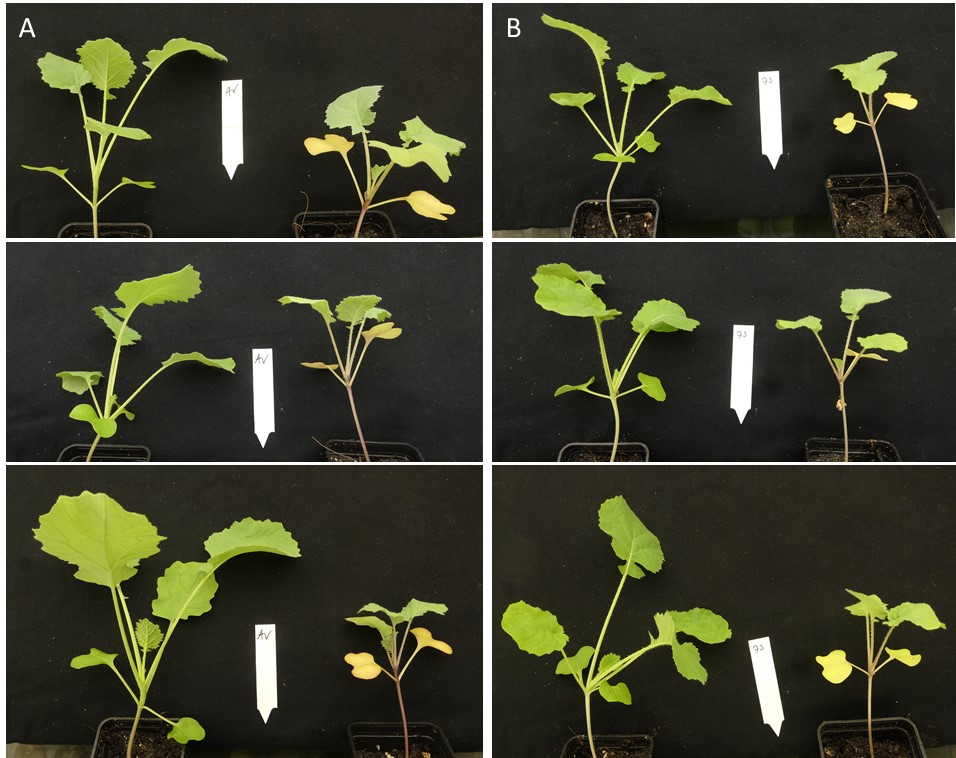
**

**Figure S9:** Pictures of *Brassica napus* plants after 14 days of waterlogging treatment on soil, one treatment with deionized water (WL, left plant on each picture), and one with 0.1% starch solution (+ starch, right plant on each picture). At the end of the treatment, plants were 29 days old. (A) Avatar; (B) Zhongshuang 9. Three representative plants from each treatment and cultivar are shown. White badge is 6 cm long.

**Figure S10:** Characterization of the two types of waterlogging treatment on soil, one with deionized water (WL), and one with 0.1% starch solution (+ starch). Treatment was done with *Brassica napus* plants for up to 14 days. At the start of treatment, plants were 15 days old. **(A)** Oxygen content in the solution. **(B)** Oxidation-reduction potential (ORP) in the solution. **(C)** pH value in the solution. **(D)** Conductivity in the solution. Data are means +/- SD of 3 treatments per type with 15 plants in each treatment. Stars indicate significant differences between water and starch solution at each time point (T-Test, * p < 0.05; ** p < 0.01).

# Supplementary Tables

Please see separate Excel files

**Table S1:** RNA sequencing statistics.

**Table S2:** RNA sequencing data presented in this publication.

**Table S3:** GO term enrichment analysis of differentially expressed transcripts.

**Table S4:** Statistics for enrichment of affected genes (hypergeometric distributions).

**Table S5:** Heatmaps for metabolism-related expression data.

**Table S6:** Heatmaps for hormone-related expression data.

**Table S7:** Comparison of the expression data from this publication to data from Guo et al. (2020) and Li et al. (2021).

**Table S8:** Expression data of candidate genes from Zou et al. (2015) and Ding et al. (2020).
